# Chemistry of a Nitrosyl Ligand κ:η-Bridging a Ditungsten Center: Rearrangement and N–O Bond Cleavage Reactions

**DOI:** 10.1021/acs.inorgchem.2c02216

**Published:** 2022-09-15

**Authors:** M. Angeles Alvarez, M. Esther García, Daniel García-Vivó, Ana M. Guerra, Miguel A. Ruiz, Larry R. Falvello

**Affiliations:** † Departamento de Química Orgánica e Inorgánica/IUQOEM, Universidad de Oviedo, Oviedo E33071, Spain; ‡ Instituto de Nanociencia y Materiales de Aragón, Departamento de Química Inorgánica, CSIC, Universidad de Zaragoza, Zaragoza E-50009, Spain

## Abstract

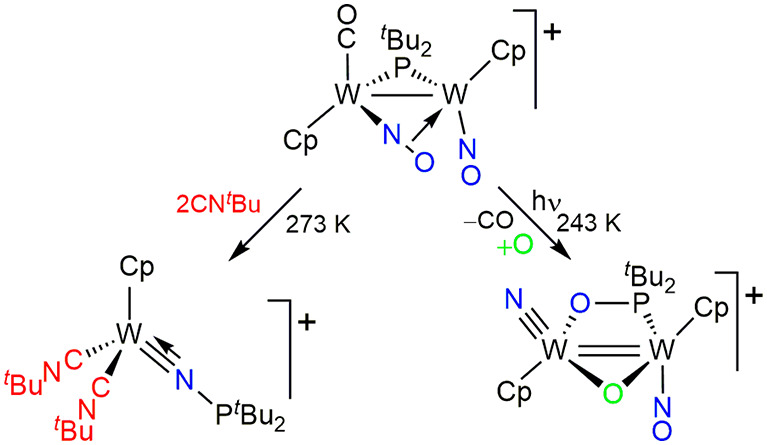

The novel nitrosyl-bridged complex [W_2_Cp_2_(μ-P^*t*^Bu_2_)(μ-κ:η-NO)(CO)(NO)](BAr_4_) [Ar = 3,5-C_6_H_3_(CF_3_)_2_] was prepared in a multistep procedure starting from the hydride [W_2_Cp_2_(μ-H)(μ-P^*t*^Bu_2_)(CO)_4_] and involving the new complexes [W_2_Cp_2_(μ-P^*t*^Bu_2_)(CO)_4_](BF_4_), [W_2_Cp_2_(μ-P^*t*^Bu_2_)(CO)_2_(NO)_2_](BAr_4_), and [W_2_(μ-κ:η^5^-C_5_H_4_)Cp(μ-P^*t*^Bu_2_)(CO)(NO)_2_] as intermediates, which follow from reactions with HBF_4_·OEt_2_, NO, and Me_3_NO·2H_2_O, respectively. The nitrosyl-bridged cation easily added chloride upon reaction with [N(PPh_3_)_2_]Cl, with concomitant NO rearrangement into the terminal coordination mode, to give [W_2_ClCp_2_(μ-P^*t*^Bu_2_)(CO)(NO)_2_], and underwent N–O and W–W bond cleavages upon the addition of CN^*t*^Bu to give the mononuclear phosphinoimido complex [WCp(NP^*t*^Bu_2_)(CN^*t*^Bu)_2_](BAr_4_). Another N–O bond cleavage was induced upon photochemical decarbonylation at 243 K, which gave the oxo- and phosphinito-bridged nitrido complex [W_2_Cp_2_(N)(μ-O)(μ-OP^*t*^Bu_2_)(NO)](BAr_4_), likely resulting from a N–O bond cleavage step following decarbonylation.

## Abstract 2

The π binding of the NO ligand in the cation [W_2_Cp_2_(μ-P^*t*^Bu_2_)(μ-κ:η-NO)(CO)(NO)]^+^ facilitates the addition of ligands with concomitant rearrangement of the bridging nitrosyl into the terminal coordination mode and also facilitates cleavage of the N−O bond of that ligand at low temperature possibly in two different ways: either through the oxidative addition of this ligand to the dimetal center or through deoxygenation by another ligand.

Nitric oxide (NO) is a multifaceted molecule able to bind metal atoms in both high and low oxidation states with very diverse coordination modes, which makes the chemistry of nitrosyl complexes a research area of great academic interest.^1^ This simple molecule also has relevant biological activities at low doses (neurotransmission, regulation of blood pressure, tumorigenic activity, etc.),^2^ and some nitrosyl complexes can actually be designed as drugs releasing NO in a controlled way for therapeutic purposes.^3^ In contrast, the presence of NO in air at high levels has undesired consequences (toxicity, greenhouse effect, destruction of stratospheric ozone, etc.), which makes it an important air pollutant requiring catalytic, metal-mediated abatement.^4^ The latter often involves cleavage of the strong N–O bond of this molecule while interacting with one or more metal atoms at the surface of solid catalysts. Such elemental reactions thus become fundamental processes to be studied in search of potential improvements in the catalysis for NO abatement. This is why we have been studying for some time the chemistry of binuclear nitrosyl complexes bearing different types of unsaturation (coordinative, electronic, or both) because these are molecular systems potentially able to activate (weaken) and eventually cleave the N–O bond of nitric oxide at a dimetal site.^5−7^

The nitrosyl ligand is known to bind two metal atoms in four distinct ways (**A** to **D** in Chart 1). In the more common N:N bridging (or semibridging) mode (**A**), the ligand provides the dimetal center with three electrons and is somewhat activated toward cleavage of its N–O bond, which is weakened with respect to the bond in terminal linear nitrosyls in mononuclear complexes. The coordination of type **B** involves additional binding of the oxygen atom to a second metal atom via lone electron pairs of oxygen and should not modify much the strength of the N–O interaction. In contrast, the bent (**C**) or linear (**D**) κ:η modes involve coordination to the second metal atom via a π(N–O) bonding orbital, and this would be expected to significantly weaken that bond. Unfortunately, only a couple of examples of these coordination modes are currently known,^8,9^ indeed displaying elongated N–O bonds of ca. 1.30 Å, and their chemistry remains unexplored. As a part of our studies on unsaturated binuclear nitrosyl complexes, we here report an efficient synthetic route for a new complex of type **D**, the cationic complex [W_2_Cp_2_(μ-P^*t*^Bu_2_)(μ-κ:η-NO)(CO)(NO)](BAr_4_) (Ar = 3,5-C_6_H_3_(CF_3_)_2_) and a preliminary study of its chemical behavior. Interestingly, several N–O bond cleavage reactions can be induced at the strongly acidic dimetal site of this cation under mild conditions.

**Chart 1:**
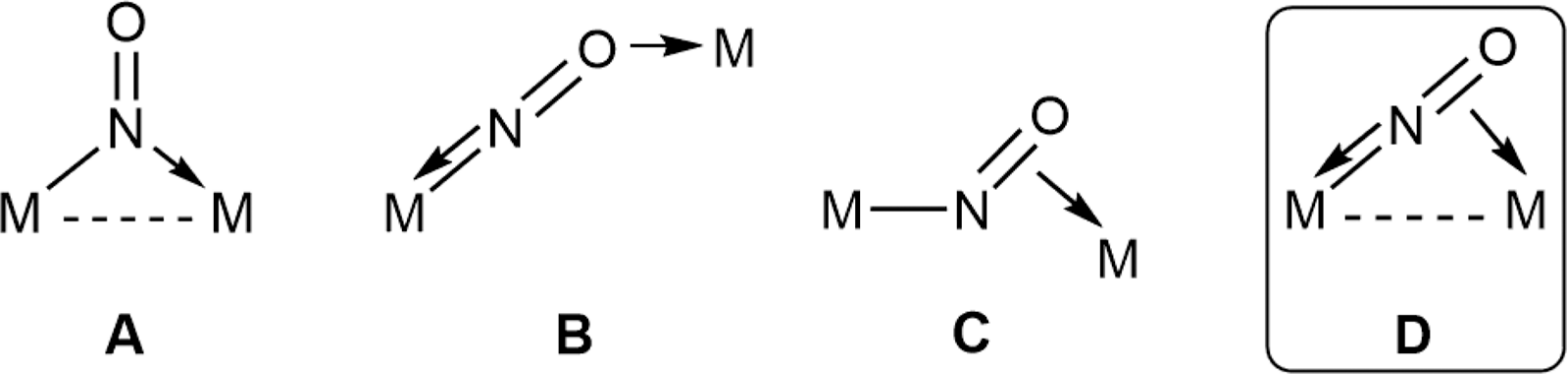
Coordination Modes of Nitrosyl Ligands in Binuclear Complexes

The mentioned κ:η nitrosyl-bridged complex was prepared through a multistep procedure starting from the corresponding hydrido-bridged complex [W_2_Cp_2_(μ-H)(μ-P^*t*^Bu_2_)(CO)_4_] (**1**) (Schemes 1 and 2). The latter was prepared as reported previously for its PCy_2_-bridged analogue.^10^ This complex was first dehydrogenated upon reaction with HBF_4_·OEt_2_, a process implemented previously for similar precursors,^11^ to selectively give the corresponding unsaturated cationic complex [W_2_Cp_2_(μ-P^*t*^Bu_2_)(CO)_4_](BF_4_) (**2**), which was not further purified. The latter was then reacted with NO (1 atm) at room temperature to give, after anion exchange with Na(BAr_4_), the electron-precise dinitrosyl complex [W_2_Cp_2_(μ-P^*t*^Bu_2_)(CO)_2_(NO)_2_](BAr_4_) (**3**) in high yield. Spectroscopic data for this cation (see the Supporting Information, SI) were comparable to those recently reported by us for the PCy_2_-bridged analogues,^5^ although now only the most symmetric isomer (with *C*_2_ symmetry) is present in solution, likely because of the superior steric pressure imposed by the bulky P^*t*^Bu_2_ ligand. Attempts to generate unsaturated derivatives of the latter cation via decarbonylation through irradiation with visible–UV light or prolonged heating in a refluxing toluene solution led only to the eventual decomposition of this complex. In contrast, decarbonylation took place easily even at 273 K upon reaction with Me_3_NO (either hydrated or dehydrated) in a fluorobenzene solution, but then deprotonation of a cyclopentadienyl hydrogen by NMe_3_ unexpectedly occurred too, to give the neutral cyclopentadienylidene-bridged derivative [W_2_(μ-κ:η^5^-C_5_H_4_)Cp(μ-P^*t*^Bu_2_)(CO)(NO)_2_] (**4**) as a unique organometallic product, which was isolated in good yield (60%) after chromatographic workup. An X-ray study on the molybdenum analogue of **4** confirmed the coordination mode of the cyclopentadienilydene ligand in these products,^12^ as is also evident from the spectroscopic data of **4** (see the SI).

**Scheme 1:**
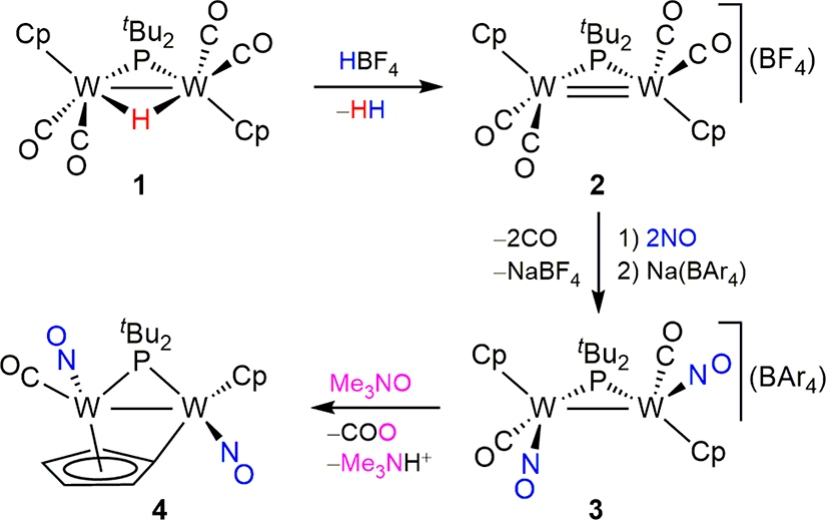
Synthesis of Compound **4**

**Scheme 2:**
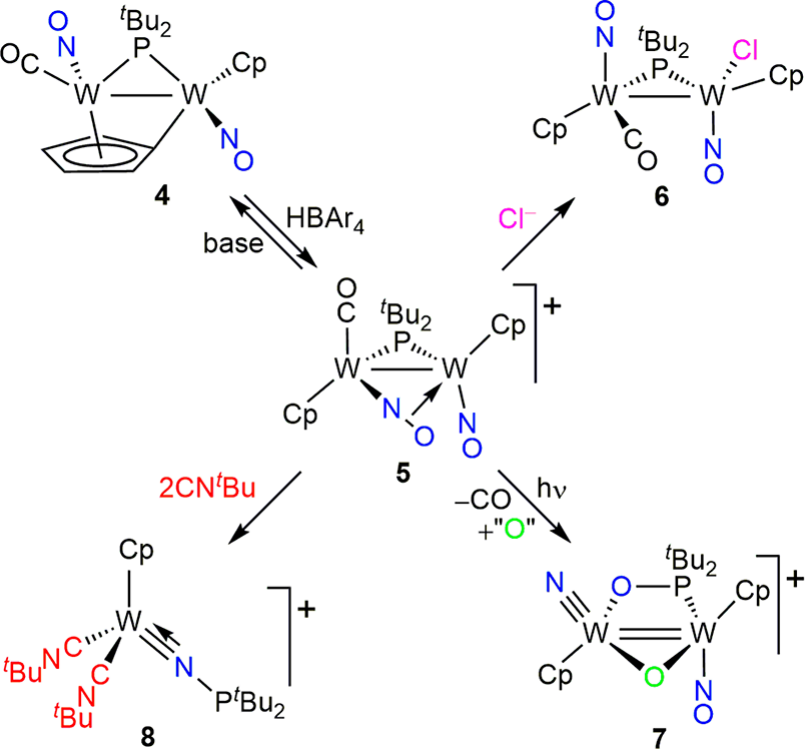
Reactivity of Compound **5** The counterion is (BAr_4_)^−^ for all cations.

We have shown previously that bridging cyclopentadienylidene ligands can be protonated at the bridgehead carbon atom to regenerate cyclopentadienyl ligands,^13^ and this is also the case of compound **4**. Indeed, the latter reacts instantaneously with [H(OEt_2_)_2_](BAr_4_) in a dichloromethane solution to give the cyclopentadienyl derivative [W_2_Cp_2_(μ-P^*t*^Bu_2_)(μ-κ:η-NO)(CO)(NO)](BAr_4_) (**5**), in a process that can be reversed upon reaction with different bases (Scheme 2). The spectroscopic data for **5** denoted the presence of terminal CO and NO ligands, but the coordination mode of the second nitrosyl ligand was not obvious. An X-ray study revealed that the latter displays the unusual linear κ:η bridging mode (type **D**), with it being strongly bound to one metal via the nitrogen atom [W2–N3 = 1.823(4) Å], while π binding the second metal atom through its N–O bond [W1–N3 = 2.191(4) Å; W1–O3 = 2.138(3) Å], which then becomes significantly elongated [N3–O3 = 1.271(5) Å] and presumably debilitated, in agreement with its very low N–O stretching frequency (1366 cm^–1^). The coordination mode of the NO ligand found in compound **5** was relatively unexpected because previous DFT studies on the decarbonylation products of the neutral complexes [W_2_Cp_2_(μ-PCy_2_)(CO)_3_(NO)] revealed that carbonyl is a better-suited ligand than NO for bridging two metal atoms in a linear κ:η fashion.^14^ In fact, density functional theory (DFT) calculations on the cation in **5** (Figure 1) and some possible isomers revealed that a linear κ:η-CO-bridged isomer would have the lowest energy, with the actual ordering found, when the nature and coordination mode of the bridging ligand are changed, being μ-κ:η-CO (0) < μ-κ:η-NO (+16) < μ-NO (+62) (relative Gibbs free energies at 298 K in kJ/mol; see the SI). Therefore, we conclude that **5** is a kinetic product. However, refluxing toluene solutions of this compound resulted in no detectable rearrangement.

**Figure 1:**
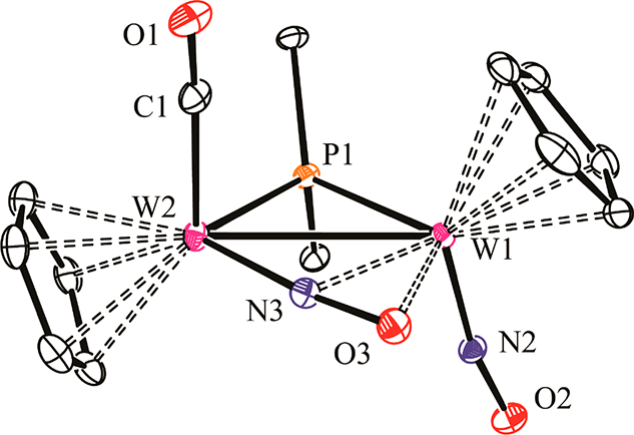
ORTEP diagram (30% probability) of the cation in **5**, with hydrogen atoms and methyl groups omitted. Selected bond lengths (Å) and angles (deg): W1–W2 = 3.0978(3); W1–N2 = 1.771(4); W2–N3 = 1.823(4); W1–N3 = 2.191(4); W1–O3 = 2.138(3); N2–O2 = 1.205(5); N3–O3 = 1.271(5); W2–N3–O3 = 171.3(3).

Preliminary studies on the reactivity of **5** (Scheme 2) revealed that the π binding of the NO ligand can be removed upon the addition of simple donors, with concomitant rearrangement of the latter to terminal coordination, as found for κ:η-CO-bridged complexes. Thus, compound **5** reacts smoothly with CO (1 atm) at 348 K in a 1,2-dichloroethane solution to regenerate compound **3** selectively. It also easily adds chloride upon reaction with [N(PPh_3_)_2_]Cl at room temperature to give the neutral derivative [W_2_ClCp_2_(μ-P^*t*^Bu_2_)(CO)(NO)_2_] (**6**), with the chloride ligand in a cisoid positioning with respect to the phosphorus atom [P–W–Cl = 84.45(5)°], while the carbonyl ligand adopts a transoid arrangement [P–W–CO = 105.4(2)°] with incipient semibridging geometry [W–W–CO = 61.9(2)°; see the SI], also reflected in an anomalously low C–O stretching frequency in solution (1903 cm^–1^). The addition of donor molecules, however, is limited by the high Brönsted acidity of the cation in **5**, which turned out to be deprotonated by conventional phosphines and amines or even upon dissolution of the complex in tetrahydrofuran, to give the cyclopentadienylidene precursor **4** quantitatively.

The carbonyl ligand in **5** can be removed photochemically at 243 K, in a process that also triggers cleavage of the N–O bond of one of the nitrosyl ligands and abstraction of an additional oxygen atom to give the oxo- and phosphinito-bridged nitrido complex [W_2_Cp_2_(N)(μ-O)(μ-OP^*t*^Bu_2_)(NO)](BAr_4_) (**7**; Figure 2).^15^ Presumably, this N–O bond cleavage would follow decarbonylation to give first an oxo nitrido intermediate, as previously observed for some bridging nitrosyl ligands of type **A** at M_2_^7a^ or M_2_M′ centers (M = Mo, W; M′ = Mn, Re).^6,16^ This would be followed by insertion of the oxo ligand into a W–P bond to generate the bridging phosphinito ligand and the addition of an extra oxygen atom (likely from trace dioxygen in the solution) to the unsaturated dimetal center thus generated.^17^

**Figure 2:**
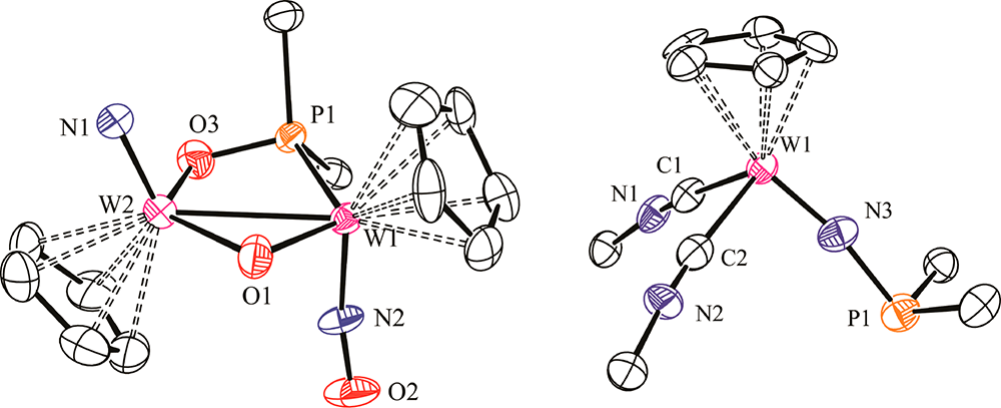
ORTEP diagrams (30% probability) of the cations in compounds **7** (left) and **8** (right), with hydrogen atoms and methyl groups omitted. Selected bond lengths (Å) and angles (deg) for **7**: W1–W2 = 2.823(1); W1–P1 = 2.47(1); W1–O1 = 1.97(2); W2–O1 = 2.29(3); W2–N1 = 1.70(2); W2–O3 = 1.98(2). Selected bond lengths (Å) and angles (deg) for **8**: W–C1 = 2.06(1); W–C2 = 2.12(2); W–N3 = 1.72(2); W–N3–P = 170(1).

The exact nature and geometry of compound **7** could only be established through a crystallographic study, even if the precision in the geometrical parameters was modest, because the asymmetric cation lies on a plane of symmetry in the unit cell (a case of whole-body disorder; see the SI). The terminal nitrido ligand in the cation is strongly bound to a tungsten atom, as revealed by the short W2–N1 length of 1.70(2) Å^18^ and the presence of a relatively energetic W–N stretch of 962 cm^–1^ in the IR spectrum. The bridging oxo ligand displays W–O lengths above ca. 2.0 Å, which is indicative of modest π bonding.^16^ As a result, the cation can be considered to be a 32e complex, for which a double intermetallic bond should be proposed according to the 18e rule. This is consistent with the intermetallic distance of 2.823(1) Å in **7**, much shorter than those measured for the electron-precise complexes **5** and **6** [3.0978(3) and 3.1812(4) Å, respectively].

A different and unexpected N–O bond cleavage takes place in the reaction of **5** with CN^*t*^Bu. This reaction proceeds at 273 K, even when 1 equiv of isocyanide is used, to give, as the major phosphorus-containing species, the mononuclear bis(isocyanide) complex [WCp(NP^*t*^Bu_2_)(CN^*t*^Bu)_2_](BAr_4_) (**8**), which displays a four-electron donor linear phosphinoimido ligand with a very short W–N separation of 1.72(2) Å (Figure 2).^19−21^ No intermediates were identified in this obviously multistep reaction, and further studies are now in progress to determine whether this reaction involves the oxidative addition of the κ:η-bridging nitrosyl ligand to the dimetal center, as proposed for **7**, or rather follows from a direct deoxygenation reaction by isocyanide or carbonyl ligands, thus paralleling to some extent the reactions of the κ:κ-nitrosyl-bridged complex [Mo_2_Cp_2_(μ-PCy_2_)(μ-NO)(NO)_2_] with phosphites or CO.^5^

In summary, we have implemented an efficient preparative route for a new tungsten complex bearing a nitrosyl ligand in the rare linear κ:η bridging coordination mode, by starting from the readily available P^*t*^Bu_2_-bridged precursor **1**, and involving the new complexes **2**–**4** as intermediate species. The reactivity of this nitrosyl-bridged complex indicates that π binding of the NO ligand to the second metal atom has two chemical effects not identified previously: (a) it facilitates the addition of ligands with concomitant rearrangement of the bridging nitrosyl into the terminal coordination mode, as found for linear κ:η-CO-bridged complexes, and (b) it facilitates cleavage of the N–O bond of that ligand, possibly in two different ways: either through the oxidative addition of the ligand to the dimetal center or through deoxygenation by another ligand. None of these bond cleavage processes is known for the carbonyl ligand in the related κ:η bridging mode.

## Supporting Information Available

Preparative procedures, spectroscopic and microanalytical data for new compounds, crystallographic data, and results of DFT calculations (PDF)
Cartesian coordinates for all computed species (XYZ)

## Supplementary Material

ic2c02216_si_001.pdf

ic2c02216_si_002.xyz
