# Supplementary material for: Chemistry of a Nitrosyl Ligand κ:η-Bridging a Ditungsten Center: Rearrangement and N–O Bond Cleavage Reactions
Source: Inorg Chem. 2022 Sep 15;61(38):14929–33. doi: 10.1021/acs.inorgchem.2c02216 (PMC9516685; doi:10.1021/acs.inorgchem.2c02216)
Supplement: Supplementary file 1 — ic2c02216_si_001.pdf [file ic2c02216_si_001.pdf]

Supporting Information

Chemistry of a Nitrosyl Ligand κ:η-Bridging a Ditungsten Center: Rearrangement and N–O Bond Cleavage Reactions

M. Angeles Alvarez[a], M. Esther García[a], Daniel García-Vivó[a]*, Ana M. Guerra[a], Miguel A. Ruiz[a]*, and Larry R. Falvello[b]

[a]*Departamento de Química Orgánica e Inorgánica/IUQOEM, Universidad de Oviedo, E-33071 Oviedo, Spain.*

[b]*Instituto de Nanociencia y Materiales de Aragón (INMA), Departamento de Química Inorgánica, CSIC - Universidad de Zaragoza, E-50009, Zaragoza, Spain.*

*E-mail: garciavdaniel@uniovi.es (D.G.V.), mara@uniovi.es (M.A.R).

Experimental Section

General Procedures and Starting Materials. All manipulations and reactions were carried out under an argon (99.995%) atmosphere using standard Schlenk techniques. Solvents were purified according to literature procedures, and distilled prior to use.[1] Compounds $[W_2Cp_2(CO)_6]$,[2] and $[H(OEt_2)_2](BAr_4)$,[3] (Ar = 3,5-$C_6H_3(CF_3)_2$), were prepared as described previously, and all other reagents were obtained from the usual commercial suppliers and used as received, unless otherwise stated. Petroleum ether refers to that fraction distilling in the range 338-343 K. Photochemical experiments were performed using jacketed Pyrex Schlenk tubes cooled by a closed 2-propanol circuit kept at the desired temperature with a cryostat. A 400 W medium-pressure mercury lamp placed ca. 2 cm away from the Schlenk tube was used for these experiments. Filtrations were carried out through diatomaceous earth unless otherwise stated. Chromatographic separations were carried out using jacketed columns refrigerated by a closed 2-propanol circuit kept at the desired temperature with a cryostat. Commercial silica gel (230-400 mesh) and aluminum oxide (activity I, 70-290 mesh) were degassed under vacuum prior to use. The latter was mixed under argon with the appropriate amount of water to reach activity IV. IR stretching frequencies of CO and NO ligands were generally measured in solution (using CaF_2 windows), are referred to as ν(XO) and are given in wave numbers (cm^{-1}). Nuclear magnetic resonance (NMR) spectra were recorded at 295 K unless otherwise stated. Chemical shifts (δ) are given in ppm, relative to internal tetramethylsilane (^{1}H, ^{13}C), or external 85% aqueous H_3PO_4 solutions (^{31}P). Coupling constants (J) are given in hertz.

Preparation of $[W_2Cp_2(\mu\text{-H})(\mu\text{-P}^t\text{Bu}_2)(CO)_4]$ (1). A diglyme solution (15 mL) of $[W_2Cp_2(CO)_6]$ (0.750 g, 1.126 mmol) was refluxed for 7 h with a gentle N_2 purge to give a red-brown solution of $[W_2Cp_2(CO)_4]$, which was filtered with a canula. Neat PH^tBu_2 (313 μL, 1.690 mmol) was then added, and the mixture was stirred at 353 K for 16 h to give a red-brown solution. The solvent was then removed under vacuum, the residue was extracted with dichloromethane/petroleum ether (1/3), and the extracts were chromatographed on silica gel at 288 K. Elution with dichloromethane/petroleum ether (1/2) gave a red fraction yielding, upon removal of solvents, compound **1** as a red microcrystalline solid (0.560 g, 66%). Anal. Calcd for $C_{22}H_{29}O_4PW_2$: C, 34.95; H, 3.87. Found: C, 34.65; H, 3.58. ν(CO) (CH_2Cl_2): 1941 (w, sh), 1921 (vs), 1840 (s). $^{31}P\{^1H\}$ NMR (121.51 MHz, CD_2Cl_2): δ 183.1 (s, J_{PW} = 181). ^{1}H NMR (300.13 MHz, CD_2Cl_2): δ 5.45 (s, 10H, Cp), 1.38 (s, br, 18H, ^{t}Bu), –16.29 (d, J_{HP} = 25, J_{HW} = 40, μ-H). $^{13}C\{^1H\}$ NMR (100.63 MHz, CD_2Cl_2): δ 235.5 (d, J_{CP} = 20 Hz, WCO), 226 (s, WCO), 89.7 (s, Cp), 42.4 [d, J_{CP} = 14, $C^1(^t\text{Bu})$], 32.9 [s, $C^2(^t\text{Bu})$].

Preparation of $[W_2Cp_2(\mu\text{-P}^t\text{Bu}_2)(CO)_4]BF_4$ (2). Neat $HBF_4\cdot OEt_2$ (162 μL, 1.190 mmol) was added to a dichloromethane solution (20 mL) of compound **1** (0.450 g, 0.595 mmol), and

the mixture was stirred at room temperature for 1 h to give a black solution that was filtered. The solvent was then removed from the filtrate under vacuum, the residue was washed with diethyl ether (4 x 10 mL), and dried under vacuum to give essentially pure compound **2** as a black powder, which was used without further purification (0.460 g, 92%). ν(CO) (CH_2Cl_2): 2002 (m), 1952 (vs), 1935 (s), 1900 (w). $^{31}P\{^1H\}$ NMR (121.51 MHz, CD_2Cl_2): δ 170.6 (s, J_{PW} = 214). ^1H NMR (400.13 MHz, CD_2Cl_2): δ 6.03 (s, 10H, Cp), 1.34 (d, J_{HP} = 16, ^{t}Bu). $^{13}C\{^1H\}$ NMR (100.63 MHz, CD_2Cl_2): δ 216.6 (br, WCO), 95.1 (s, Cp), 48.9 [d, J_{CP} = 18, C^1(^{t}Bu)], 33.5 [d, J_{CP} = 4, C^2(^{t}Bu)].

Preparation of [$W_2Cp_2(\mu$-$P^tBu_2)(CO)_2(NO)_2$](BAr_4) (3). Nitric oxide (5% in N_2) was gently bubbled through an stirred 1,2-dichloroethane solution (15 mL) of compound **2** (0.460 g, 0.546 mmol) for 1.5 h at room temperature, to give an orange solution. Solid Na(BAr_4) (0.484 g, 0.546 mmol) was then added, and the mixture was stirred for 10 min, then filtered. Removal of the solvent under vacuum gave essentially pure compound **3** as a yellow solid (0.800 g, 90%), ready for further use. Additional purification can be achieved upon chromatography on alumina at 288 K. To this purpose, the crude product is dissolved in dichloromethane/petroleum ether (3/1) and eluted with neat dichloromethane to give, after removal of solvents, compound **3** as a yellow microcrystalline solid (0.560 g, 63%). Anal. Calcd for $C_{52}H_{40}BF_{24}N_2O_4PW_2$: C, 38.50; H, 2.49; N, 1.53. Found: C, 38.84; H, 2.27; N, 1.78. ν(XO) (CH_2Cl_2): 2036 (m, sh), 2022 (vs), 1667 (s). $^{31}P\{^1H\}$ NMR (121.49 MHz, CD_2Cl_2): δ 240.9 (s, J_{PW} = 177). ^1H NMR (400.13 MHz, CD_2Cl_2): δ 7.72 (s, br, 8H, Ar), 7.56 (s, 4H, Ar), 5.97 (s, 10H, Cp), 1.36 (d, J_{HP} = 16, ^{t}Bu). $^{13}C\{^1H\}$ NMR (100.63 MHz, CD_2Cl_2): δ 207.6 (d, J_{CP} = 18, WCO), 162.1 [q, J_{CB} = 51, C^1(Ar)], 135.2 [s, C^2(Ar)], 129.2 [qq, J_{CF} = 32, J_{CB} = 4, C^3(Ar)], 124.4 (q, J_{CF} = 274, CF_3), 117.3 [spt, J_{CF} = 4, C^4(Ar)], 96.9 (s, Cp), 48.7 [d, J_{CP} = 13, C^1(^{t}Bu)], 33.1 [d, J_{CP} = 3, C^2(^{t}Bu)].

Preparation of [$W_2(\mu$-κ:η^5-C_5H_4)Cp(μ-P^tBu_2)(CO)(NO)$_2$] (4). Solid $Me_3NO\cdot 2H_2O$ (0.061 g, 0.541 mmol) was added to a fluorobenzene solution (20 mL) of compound **3** (0.760 g, 0.468 mmol), and the mixture was stirred at 273 K for 3 h to give a red solution. After removal of the solvent under vacuum, the residue was extracted with dichloromethane/petroleum ether (1/1), and the extracts were chromatographed on alumina at 253 K. Elution with the same solvent mixture gave an orange fraction yielding, after removal of solvents, compound **4** as an orange microcrystalline solid (0.200 g, 59%). Anal. Calcd for $C_{19}H_{27}N_2O_3PW_2$: C, 31.26; H, 3.73; N, 3.84. Found: C, 30.98; H, 3.45; N, 3.65. ν(XO) (CH_2Cl_2): 1962 (s), 1603 (m, sh), 1576 (vs). $^{31}P\{^1H\}$ NMR (121.49 MHz, CD_2Cl_2): δ 242.6 (s, J_{PW} = 299, 197). ^1H NMR (400.13 MHz, CD_2Cl_2): δ 6.23, 6.14 (2m, 2 x 1H, C_5H_4), 5.99 (s, 5H, Cp), 5.23, 4.19 (2m, 2 x 1H, C_5H_4), 1.51 (d, J_{HP} = 13, 9H, ^{t}Bu), 1.28 (d, J_{HP} = 15, 9H, ^{t}Bu). $^{13}C\{^1H\}$ NMR (100.63 MHz, CD_2Cl_2): δ 221.6 (d, J_{CP} = 9, WCO), 172.7 [s, J_{CW} = 114, C^1(C_5H_4)], 109.0, 105.6, 97.2 [3s, CH(C_5H_4)], 95.7 (s, Cp), 89.9 [s, CH(C_5H_4)], 47.9, 43.6 [2d, J_{CP} = 13, C^1(^{t}Bu)], 33.4, 33.2 [2d, J_{CP} = 4, C^2(^{t}Bu)].

Preparation of [$W_2Cp_2(\mu$-$P^tBu_2)(\mu$-$\kappa:\eta$-NO)(CO)(NO)](BAr$_4$) (5). Solid [$H(OEt_2)_2$](BAr$_4$) (0.033 g, 0.032 mmol) was added to a dichloromethane solution (10 mL) of compound **4** (0.020 g, 0.027 mmol), and the mixture was stirred at room temperature for 5 min to give a brown solution. Removal of the solvent under vacuum and washing of the residue with petroleum ether yielded compound **5** as an essentially pure orange-brown solid (0.041 g, 95%). The orange crystals used in the X-ray study were grown through the slow diffusion layers of toluene and petroleum ether into a concentrated dichloromethane solution of the complex at 253 K. Anal. Calcd for $C_{51}H_{40}BF_{24}N_2O_3PW_2$: C, 38.42; H, 2.53; N, 1.76. Found: C, 38.15; H, 2.67; N, 1.80. ν(XO) (CH_2Cl_2): 1975 (vs), 1641 (s). ν(XO) (Nujol): 1981 (vs), 1642 (s), 1366 (m). $^{31}P\{^1H\}$ NMR (121.49 MHz, CD_2Cl_2): δ 202.7 (s, J_{PW} = 279, 245). ^1H NMR (400.13 MHz, CD_2Cl_2): δ 7.73 (s, br, 8H, Ar), 7.57 (s, 4H, Ar), 6.23, 6.08 (2s, 2 x 5H, Cp), 1.55 (d, J_{HP} = 16, 9H, ^{t}Bu), 1.35 (s, br, 9H, ^{t}Bu). $^{13}C\{^1H\}$ NMR (100.63 MHz, CD_2Cl_2): δ 227.2 (d, J_{CP} = 3, WCO), 162.2 [q, J_{CB} = 49, C^1(Ar)], 135.2 [s, C^2(Ar)], 129.3 [qq, J_{CF} = 31, J_{CB} = 3, C^3(Ar)], 124.9 (q, J_{CF} = 273, CF_3), 117.9 [spt, J_{CF} = 4, C^4(Ar)], 102.0, 94.7 (2s, Cp), 48.8, 48.5 [2d, J_{CP} = 13, C^1(^{t}Bu)], 34.0 [d, J_{CP} = 4, C^2(^{t}Bu)], 33.5 [s, C^2(^{t}Bu)].

Preparation of [$W_2ClCp_2(\mu$-$P^tBu_2)(CO)(NO)_2$] (6). Solid [$N(PPh_3)_2$]Cl (0.025 g, 0.043 mmol) was added to a dichloromethane solution (5 mL) of compound **5**, prepared in situ from 0.030 g of compound **4** (0.041 mmol), and the mixture was stirred for 1 h to give a red solution. After removal of the solvent under vacuum, the residue was extracted with dichloromethane/petroleum ether (1/2) and the extracts were chromatographed on alumina at 288 K. Elution with dichloromethane/petroleum ether (1/1) gave an orange fraction yielding, after removal of solvents, compound **6** as an orange-brown solid (0.025 g, 80%). The crystals used in the X-ray study were grown through the slow diffusion of layers of toluene and petroleum ether into a concentrated dichloromethane solution of the complex at 253 K. Anal. Calcd for $C_{19}H_{28}ClN_2O_3PW_2$: C, 29.77; H, 3.68; N, 3.65. Found: C, 29.44; H, 3.47; N, 3.60. ν(XO) (CH_2Cl_2): 1903 (m), 1621 (s, sh), 1604 (vs). $^{31}P\{^1H\}$ NMR (121.50 MHz, CD_2Cl_2): δ 177.6 (s, J_{PW} = 278, 247). ^1H NMR (400.13 MHz, CD_2Cl_2): δ 5.89, 5.87 (2s, 2 x 5H, Cp), 1.46 (d, J_{HP} = 15, 9H, ^{t}Bu), 1.30 (d, J_{HP} = 14, 9H, ^{t}Bu). $^{13}C\{^1H\}$ NMR (100.63 MHz, CD_2Cl_2): δ 230.7 (d, J_{CP} = 5, WCO), 106.6, 95.1 (2s, Cp), 46.7, 42.6 [2d, J_{CP} = 15, C^1(^{t}Bu)], 33.6 [d, J_{CP} = 4, C^2(^{t}Bu)], 33.2 [d, J_{CP} = 3, C^2(^{t}Bu)].

Preparation of [$W_2Cp_2(N)(\mu$-O)(μ-OP^tBu_2)(NO)](BAr$_4$) (7). A fluorobenzene solution (10 mL) of compound **5**, prepared in situ from 0.025 g of **4** (0.034 mmol), was irradiated with visible-UV light for 1 h at 243 K while keeping a gentle N_2 (99.9995%) purge, to give a red solution which was filtered. After removal of the solvent from the filtrate under vacuum, the residue was crystallized by the slow diffusion of layers of toluene and petroleum ether into a concentrated dichloromethane solution of the product, to give compound **7** as a dark red crystalline solid, invariably contaminated with small amounts (typically ca. 15%) of the parent compound **5** (0.015g, 28%). The crystals used in the X-ray study were grown through

recrystallization of the above material by the slow diffusion of layers of diethyl ether and petroleum ether into a concentrated dichloromethane solution of the product at 253 K. Selected IR data (crystalline sample, ATR): 1576 [m, ν(NO)], 962 [m, ν(WN)]. $^{31}P\{^1H\}$ NMR (121.50 MHz, CD_2Cl_2): δ 132.7 (s, J_{PW} = 345, 25). ^1H NMR (300.09 MHz, CD_2Cl_2): δ 7.72 (s, br, 8H, Ar), 7.56 (s, 4H, Ar), 6.58, 6.09 (2s, 2 x 5H, Cp), 1.40 (d, J_{HP} = 14, 9H, ^{t}Bu), 1.25 (d, J_{HP} = 15, 9H, ^{t}Bu).

Preparation of [WCp(NP^{t}Bu$_2$)(CN^{t}Bu)$_2$](BAr$_4$) (8). Neat CN^{t}Bu (12 μL, 0.106 mmol) was added to a dichloromethane solution (10 mL) of compound **5**, prepared in situ from 0.025 g of **4** (0.034 mmol), and the mixture was stirred at 273 K for 15 min to give an orange solution. After removal of the solvent under vacuum, the residue was extracted with dichloromethane and the extracts were chromatographed on alumina at 288 K. Elution with the same solvent gave an orange fraction yielding, upon removal of solvent, compound **8** as an orange microcrystalline solid (0.020 g, 41%). The crystals used in the X-ray study were grown through the slow diffusion layers of toluene and petroleum ether into a concentrated dichloromethane solution of the complex at 253 K. Anal. Calcd for $C_{55}H_{53}BF_{24}N_3PW$: C, 45.95; H, 3.72; N, 2.92. Found: C, 45.59; H, 3.72; N, 3.00. ν(CN) (CH_2Cl_2): 2158 (vs), 2122 (s). $^{31}P\{^1H\}$ NMR (121.50 MHz, CD_2Cl_2): δ 137.1 (s, br). ^1H NMR (300.13 MHz, CD_2Cl_2): δ 7.72 (s, br, 8H, Ar), 7.57 (s, 4H, Ar), 5.62 (s, 5H, Cp), 1.52 (s, 18H, ^{t}Bu), 1.13 (d, J_{HP} = 12, 18 H, P^{t}Bu). $^{13}C\{^1H\}$ NMR (100.63 MHz, CD_2Cl_2): δ 162.2 [q, J_{CB} = 50, C^1(Ar)], 148.7 (s, WCN), 132.2 [s, C^2(Ar)], 129.3 [qq, J_{CF} = 31, J_{CB} = 3, C^3(Ar)], 125.3 (q, J_{CF} = 272, CF_3), 117.9 [spt, J_{CF} = 4, C^4(Ar)], 94.2 (s, Cp), 61.7 [s, C^1(N^{t}Bu)], 35.8 [d, J_{CP} = 27, C^1(^{t}Bu)], 30.9 [s, C^2(N^{t}Bu)], 28.1 [d, J_{CP} = 15, C^2(^{t}Bu)].

X-Ray Structure Determination of Compounds 6 and 8. Data collection for these compounds was performed at ca. 150 K on an Oxford Diffraction Xcalibur Nova single crystal diffractometer, using Cu Kα radiation. Images were collected at a 62 mm fixed crystal-detector distance using the oscillation method, with 1.0° (**6**) and 1.4° (**8**) oscillation and variable exposure time per image. Data collection strategy was calculated with the program *CrysAlis Pro CCD*,[4] and data reduction and cell refinement were performed with the program *CrysAlis Pro RED*.[4] An empirical absorption correction was applied using the *SCALE3 ABSPACK* algorithm as implemented in the program *CrysAlis Pro RED*. Using the program suite *WinGX*,[5] the structure was solved by Patterson interpretation and phase expansion using *SHELXL2018/3*, and refined with full-matrix least squares on F^2 using *SHELXL2018/3*.[6] All non-hydrogen atoms were generally refined anisotropically, except those involved in disorder, and all hydrogen atoms were geometrically placed and refined using a riding model to give the residuals shown in Table S1. For compound **6**, they were two independent molecules in the unit cell. One of the cyclopentadienyl rings was disordered but a satisfactory model could not be achieved; besides this, a C atom in another Cp ring had to be refined isotropically, this causing a B-level alert in the checkcif file. For compound **8**, the ^{t}Bu

groups of the cation and several CF_3 groups of the anion were found to be disordered. Only one of the ^{t}Bu groups and some of the CF_3 groups could be properly solved; the best solution was obtained by modeling them as disordered groups over two sites with 0.5/0.5 occupancies.

X-Ray Structure Determination of Compound 5. Data collection for this compound was performed at 100 K on a Bruker D8 Venture Photon III 14 κ-geometry diffractometer, using MoK_α radiation. The software *APEX4*[7] was used for collecting frames with the ω/ϕ scan measurement method. The *SAINT V8.40B* software was used for data reduction,[8] and a multi-scan absorption correction was applied with *SADABS-2016/2*.[9] The solution of the structure and refinements was performed as described above to give the residuals shown in Table S1. One CF_3 group of the tetraarylborate anion was disordered over two sites, satisfactorily modeled with 0.5/0.5 occupancies, and these atoms were refined isotropically to prevent their temperature factors from becoming non-positive definite. Yet, significant residual electron density remained in the vicinity of these F atoms and the W(2) atom, which caused some B- and C-level alerts in the checkcif file.

X-Ray Structure Determination of Compound 7. Single-crystal diffraction data for this compound were gathered using a Rigaku/OD Xcalibur diffractometer equipped with a Sapphire 3 detector, using MoKa radiation. The program *CrysAlisPro* was used for instrument control and for data reduction and corrections.[10]

Compound **7** crystallized in the orthorhombic system, with systematic absences consistent with either of the space groups *Pnnm* or *Pnn2*. The structure was composed of the anion BAr_4 and the tungsten-based cation (four each per unit cell) along with a void (two per cell) that accommodated disordered solvent whose nature was not identified through the diffraction data.

The bulky anion presented a largely ordered structure, except for the expected dynamic effects seen as transverse elongation of the displacement ellipsoids of the fluorine atoms for some of the CF_3 groups.

The cation, however, proved to be an example of “whole-body disorder,” in which the entire cation is disordered in place, about a crystallographic mirror plane that traverses the space in which the cation resides. The cation does not possess mirror symmetry. The resulting combined image of superposed cations required special treatment in the refinement to mitigate the effects of correlation.

Structure solution using either of the space groups revealed the severe disorder at the cation site with a largely ordered arrangement for the anion. The structure was developed fully in both space groups. The full disorder of the cation was observed in the non-centric, polar group *Pnn2*, where the disorder is not required by the symmetry elements present. Thus, with the same pattern described using both space groups, the centric group *Pnnm* was chosen for the final refinement, as it provides a fuller description of the symmetry of the average unit

cell. Refinement using *Pnnm* had better convergence properties and less severe correlation than refinement in the acentric group.

The initial structure solution, using *ShelxT*,[11] revealed a majority of the non-hydrogen atoms, but did not give a clear picture of the Cp fragments. The Cp groups were located on the basis of difference peaks following initial refinement. The Cp group at W1 was modelled with two-way disorder, in addition to the whole-body disorder of the entire cation. Not surprisingly, these two congeners and the single Cp fragment at W2 suffered from correlation and instability when freely refined. They were idealized as regular pentagons and refined as rigid groups. (Refinement as variable-metric groups also led to significant correlation.). Weak restraints were applied to the W...C(Cp) distances. Absolute restraints, *i.e.,* with numerical values for the target distances, were used, rather than relative restraints (similar values for chemically equivalent distances rather than specified target values). Specific restraints were also found to be necessary for the other severely disordered fragment of the cation, namely the $P(^tBu)_2$ group. Restraints were applied to the bonded C–C and 1,3-C···C distances of this fragment, along with restraints for the 1,3-P···C distances (anti-tilting restraints). As for the oxo and nitrido ligands, these were identified at bridging and terminal sites, respectively. Refinements with exchanged positions of these ligands (i.e. with terminal oxo and bridging nitride) led to less satisfactory thermal parameters for these atoms, and higher overall R indexes.

In contrast to the ample set of restraints applied to the Cp and ^{t}Bu fragments of the cation, which suffered severe disorder (and whose geometries are well established from prior studies), it was not considered necessary to apply geometrical restraints to the anion.

Simple rigid-bond restraints (affecting only the ΔMSDA value in the direction of a bond between a pair of atoms) were applied to the anisotropic displacement parameters of all bonded non-H atom pairs.

A solvent-accessible void was found to contain a number of difference peaks that we were unable to model sensibly in terms of possible solvent content. Near the end of refinement a solvent mask was calculated using *Platon*/*SQUEEZE*.[12] The void sits astride a crystallographic mirror plane; *Platon* thus found two voids per cell, related by symmetry, each with volume 219 $Å^3$ and with an estimated scattering content of 84 electrons.

For the final refinement, hydrogen atoms were placed at calculated positions and refined as riders, with Uiso(H) = x·Ueq(C), where x = 1.5 for methyl groups and 1.2 otherwise. The refinement was conducted using *ShelxL-2018/3*,[6b] and converged with the residuals listed in Table S1.

Crystals of compound **7** display an unusual combination of orthorhombic internal symmetry and a unit cell that is rigorously tetragonal and very nearly cubic. R(int) values are clearly in accord with Laue group *mmm* and in disagreement with the tetragonal and cubic Laue groups. Using R(merge) as defined in the package *WinGX*, we find R(merge) = 0.086 for Laue group

mmm, R(merge) = 0.517, 0.523 for *4/m* and *4/mmm*, respectively, and 0.796, 0.850 for *m(-3)* and *m(-3)m*, respectively. The R(merge) values for all of the possible trigonal and hexagonal symmetries were greater than 0.7. These values would seem to rule out pseudomerohedral twinning for this system; nevertheless, nine twin laws were tested in actual refinements. Six of the twin laws were derived by the program *COSET*,[13,14] as implemented in *WinGX*. In all cases the presence of twin domains was ruled out by the resulting population values, which were all near zero for the trial second or second and third domains.

The package *Olex2 v1.5*[15,16] was used in the early phases of the analysis for the interpretation of the results of initial structure solution.

Computational Details. DFT calculations on compound **5** and some likely isomers of it were carried out using the GAUSSIAN16 package,[17] and the M06L functional.[18] A pruned numerical integration grid (99,590) was used for all the calculations *via* the keyword Int=Ultrafine together with the empirical dispersion correction from Grimme and co-workers via the keyword GD3.[19] Effective core potentials and their associated double-ζ LANL2DZ basis set were used for W atoms.[20] The light elements (P, N, O, C and H) were described with the 6-31G* basis.[21] Geometry optimizations were performed under no symmetry restrictions, using initial coordinates derived from the closest X-ray data available. Frequency analyses were performed for all the stationary points to ensure that a minimum structure with no imaginary frequencies was achieved.

Table S1. Crystal Data for New Compounds

	**5**	**6**	**7**	**8**
mol formula	$C_{51}H_{40}BF_{24}N_2O_3PW_2$	$C_{19}H_{28}ClN_2O_3PW_2$	$C_{50}H_{40}BF_{24}N_2O_3PW_2$	$C_{55}H_{53}BF_{24}N_3PW$
mol wt	1594.33	766.55	1582.32	1437.63
cryst syst	triclinic	monoclinic	orthorhombic	orthorhombic
space group	P–1	$P2_1/c$	$Pnnm$	$Pna2_1$
radiation (λ, Å)	0.71073	1.54184	0.71073	1.54184
a, Å	13.2161(9)	18.8139(3)	17.8936(3)	25.1315(8)
b, Å	13.8985(9)	14.7833(2)	17.9982(3)	13.1764(4)
c, Å	15.4527(10)	17.0702(3)	17.8805(3)	18.7513(5)
α, deg	81.388(2)	90	90	90
β, deg	71.899(2)	112.775(2)	90	90
γ, deg	85.644(3)	90	90	90
V, Å^3	2666.2(3)	4377.59(13)	5758.47(17)	6209.4(3)
Z	2	8	4	4
calcd density, g cm^{-3}	1.986	2.326	1.825	1.538
absorp coeff, mm^{-1}	4.471	21.145	4.139	4.681
temperature, K	100.0(1)	150.3(6)	100(2)	149.8(3)
θ range (deg)	1.90-30.51	3.93-69.60	3.21-25.00	3.53-69.73
index ranges (h, k, l)	–18, 18; –19, 19; –22, 22	–22, 14; –17, 17; –14, 20	–21, 21; –14, 21; –21, 21	–30, 29; –13, 15; –18, 22
no. of reflns collected	175587	25430	60028	19171
no. of indep reflns (R_{int})	16254(0.0781)	8132(0.0494)	5237(0.0802)	8465(0.0415)
reflns with $I > 2\sigma(I)$	12487	7606	4497	7183
R indexes [data with $I > 2\sigma(I)$][a]	R_1 = 0.0366; wR_2 = 0.0845 [b]	R_1 = 0.0404; wR_2 = 0.1071[c]	R_1 = 0.0782; wR_2 = 0.1974[d]	R_1 = 0.0625; wR_2 = 0.1729[e]
R indexes (all data)[a]	R_1 = 0.0597; wR_2 = 0.0987 [b]	R_1 = 0.0434; wR_2 = 0.1101[c]	R_1 = 0.0868; wR_2 = 0.2027[d]	R_1 = 0.0727; wR_2 = 0.1874[e]
GOF	1.065	1.087	1.050	1.061
no. of restraints/params	0 / 759	0 / 512	517 /431	28 / 736
$\Delta\rho$(max., min.), eÅ^{-3}	2.893 / –1.757	2.316 / –3.562	2.590 / –1.282	2.459 / –1.930
CCDC deposition no	2181428	2181429	2181430	2181431

[a] $R = \Sigma||F_o| - |F_c|| / \Sigma|F_o|$. $wR = [\Sigma w(|F_o|^2 - |F_c|^2)^2 / \Sigma w|F_o|^2]^{1/2}$. $w = 1/[\sigma^2(F_o^2) + (aP)^2 + bP]$ where $P = (F_o^2 + 2F_c^2)/3$.
[b] a = 0.0429, b = 8.5749. [c] a = 0.0715, b = 0.0000. [d] a = 0.0770, b = 60.9844. [e] a = 0.1184, b = 11.0713.

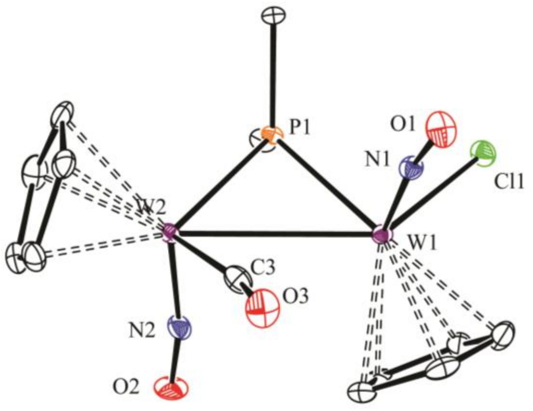

Figure S1. ORTEP diagram (30% probability) of one of the two independent molecules of compound **6** in the crystal lattice, with H atoms and Me groups omitted. Selected bond lengths (Å) and angles (º): W–W = 3.1812(4); W1–P = 2.461(2); W2–P = 2.469(2); W1–Cl1 = 2.454(2); W1–N1 = 1.774(5); W2–N2 = 1.793(6); W2–C3 = 1.994(7); W1···C3 = 2.850(7). P–W1–Cl1 = 84.45(5); P–W1–N1 = 97.5(2); P–W2–N2 = 99.7(2); P–W2–C3 = 105.4(2); Cl1–W1–N1= 92.3(2); N2–W2–C3 = 100.0(3); W1–W2–C3 = 61.9(2); W2–C3–O3 = 166.3(6).

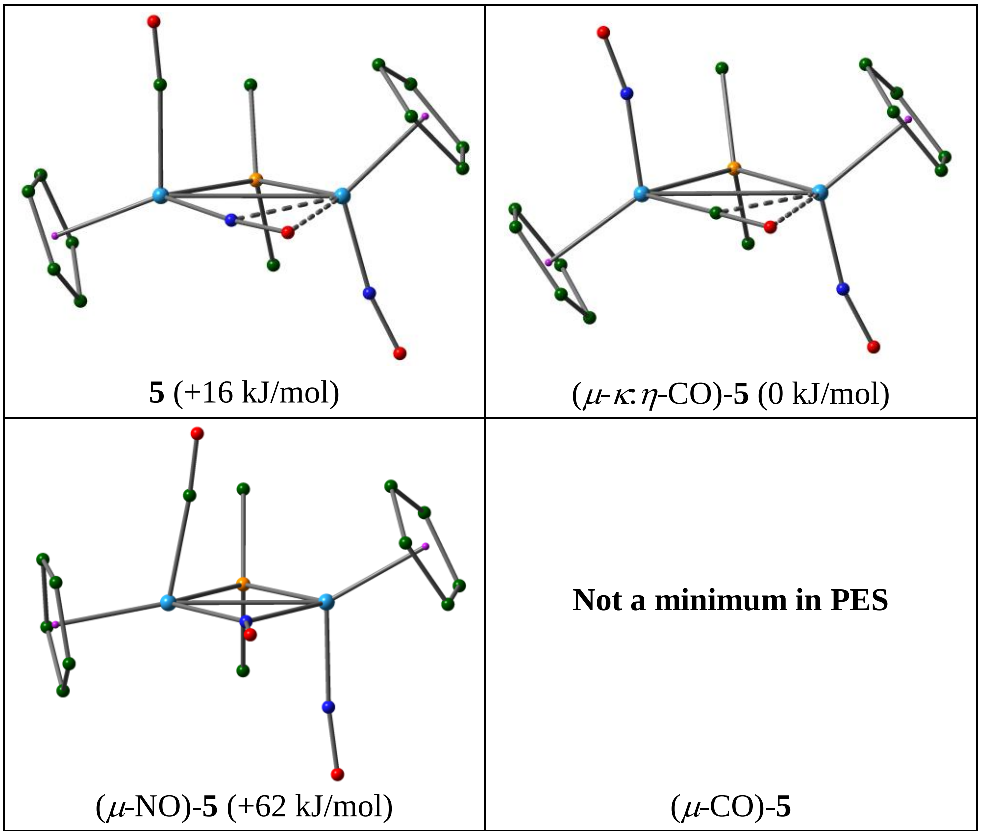

Figure S2. M06L-DFT-optimized structures of different isomers of the cation in compound **5**, with H atoms and ^{t}Bu groups (except their C^1 atoms) omitted, and relative Gibbs free energies at 298 K indicated between parentheses.

Table S2. Selected bond lengths (Å) and angles (º) for the M06L-DFT-optimized structures of different isomers of the cation in compound **5**.[a]

Parameter	**5**	(μ-NO)-**5**	(μ-κ:η-CO)-**5**
W–W	3.134	2.734	3.098
W1–N_b/C_b	1.834	2.007	1.942
W2–N_b/C_b	2.244	2.081	2.252
W2–O	2.181		2.280
W2-N	1.791	1.838	1.901
W2N–O	1.191	1.181	1.191
N_b–O_b	1.257	1.215	
C_b–O_b			1.213
W1-N_b-O_b	170.61	141.56	
W1-C_b-O_b-			169.91

[a] W1 and W2 refer to the metal atoms on the left and right at figure S2, respectively; a b subindex denotes atoms of bridging ligands.

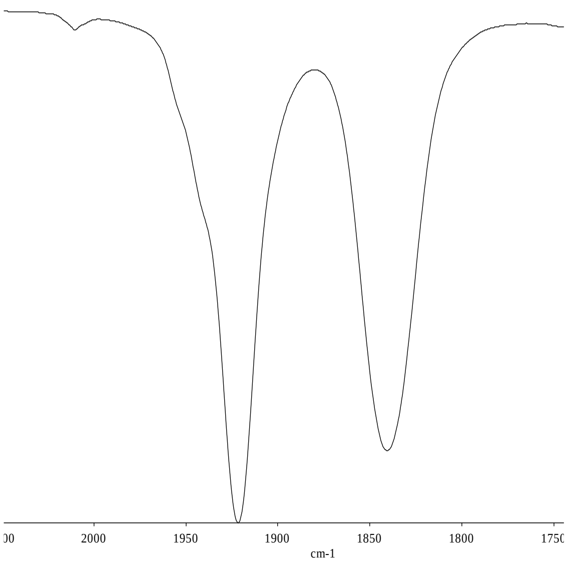

Figure S3. IR spectrum of compound **1** in dichloromethane solution.

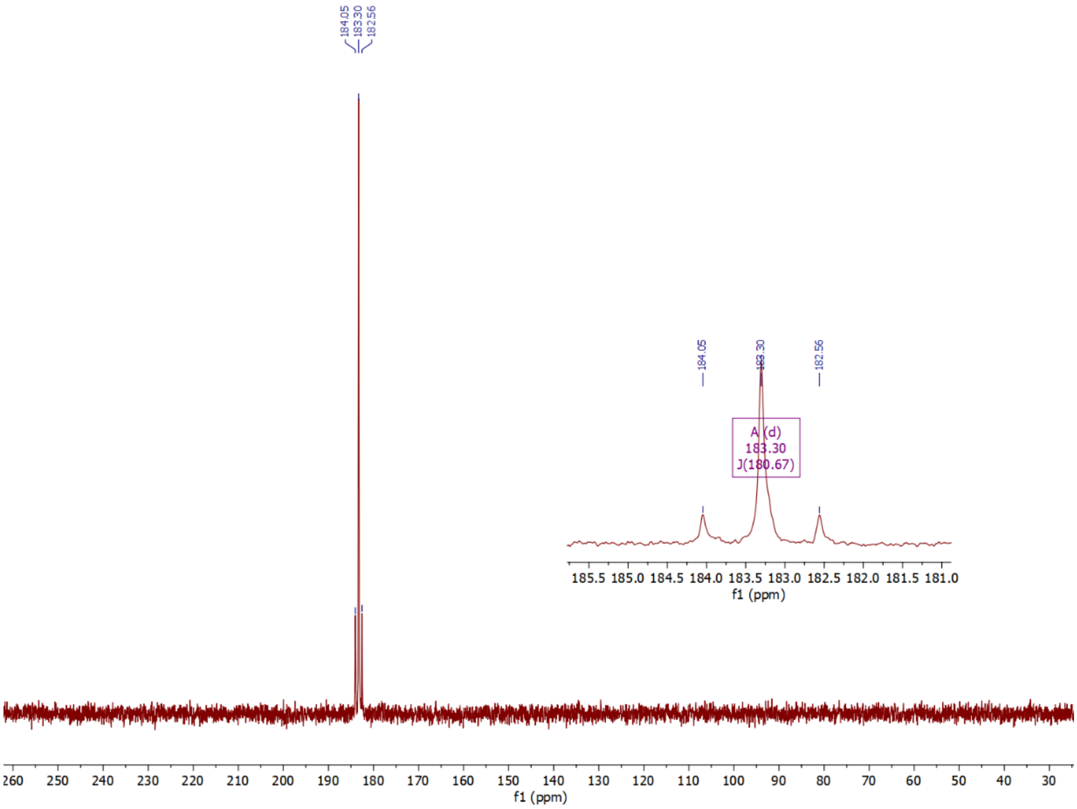

Figure S4. $^{31}P\{^1H\}$ NMR spectrum of compound **1**(CD_2Cl_2).

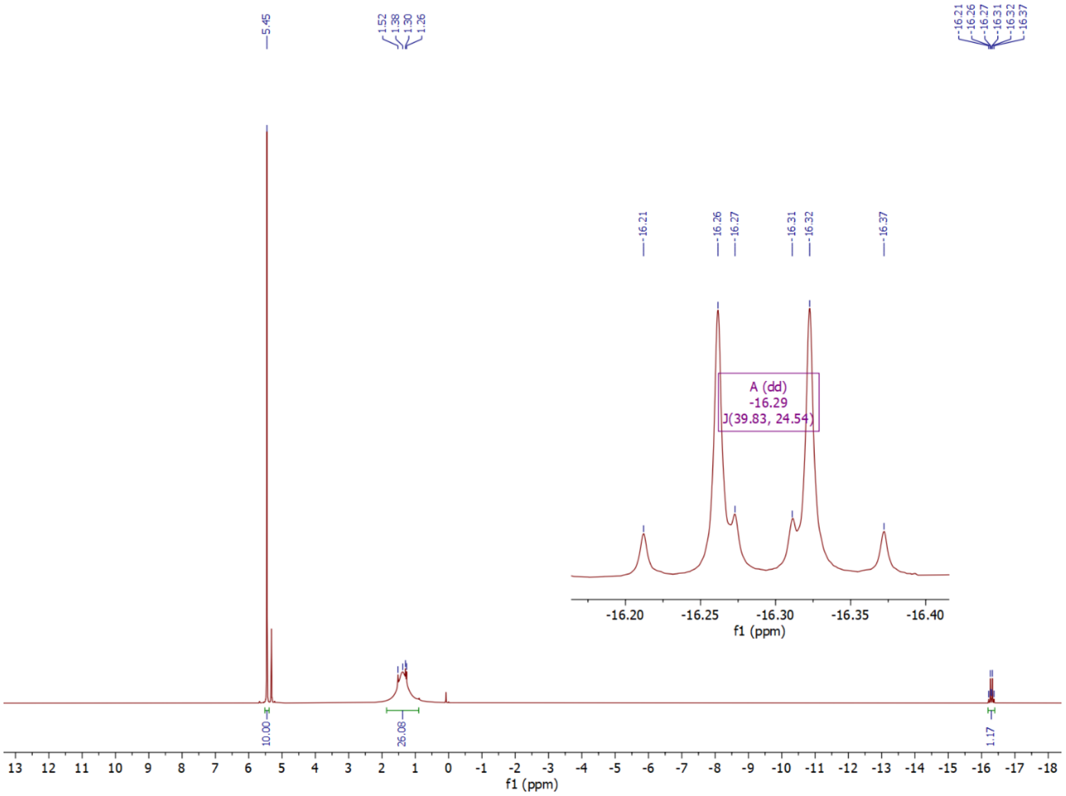

Figure S5. ^{1}H NMR spectrum of compound **1** (CD_2Cl_2).

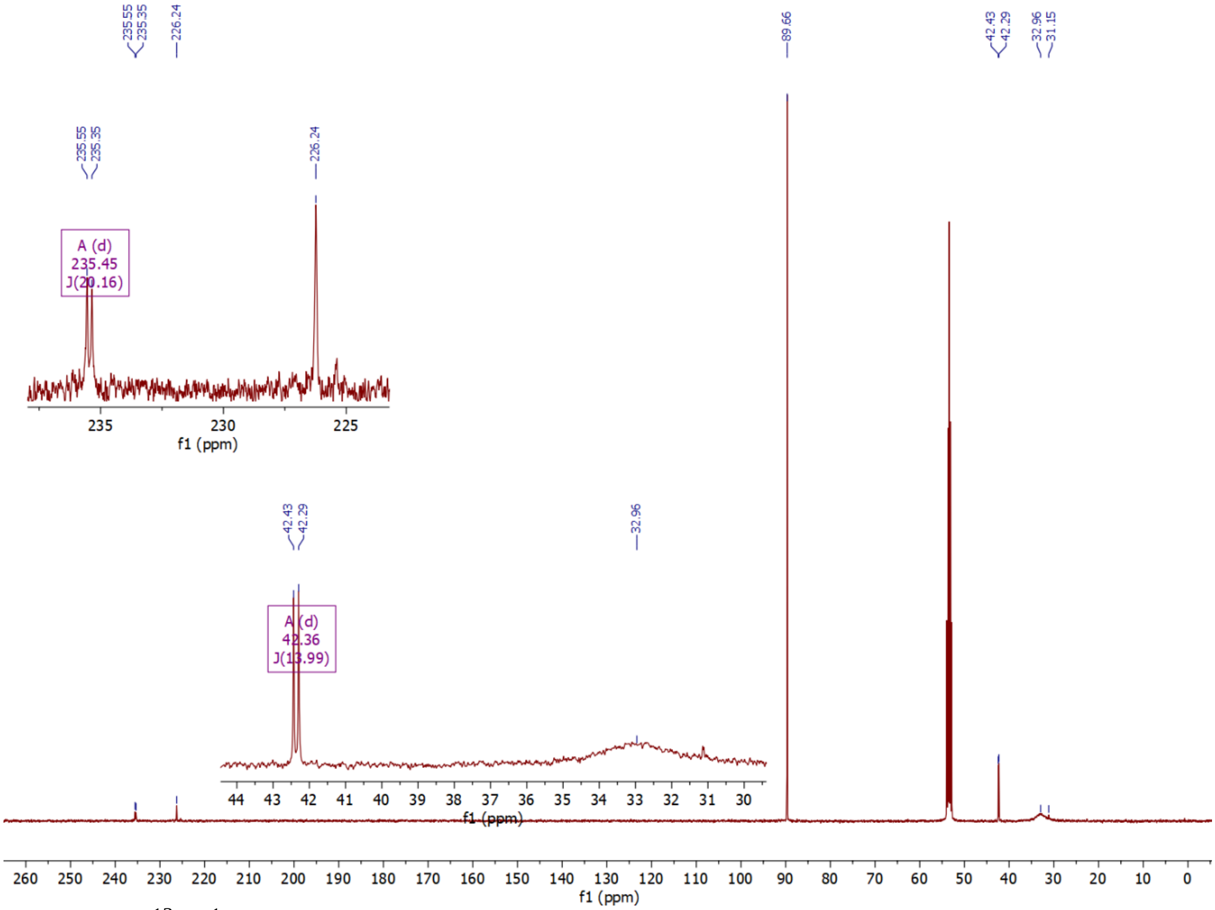

Figure S6. $^{13}C\{^{1}H\}$ NMR spectrum of compound **1** (CD_2Cl_2).

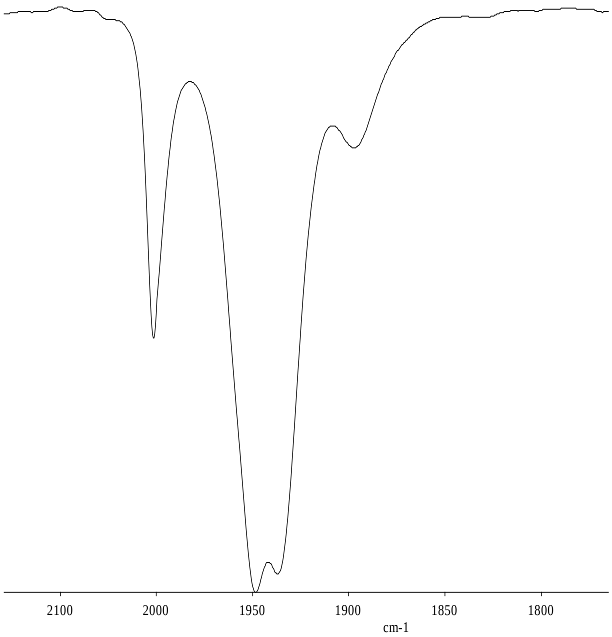

Figure S7. IR spectrum of compound **2** in dichloromethane solution.

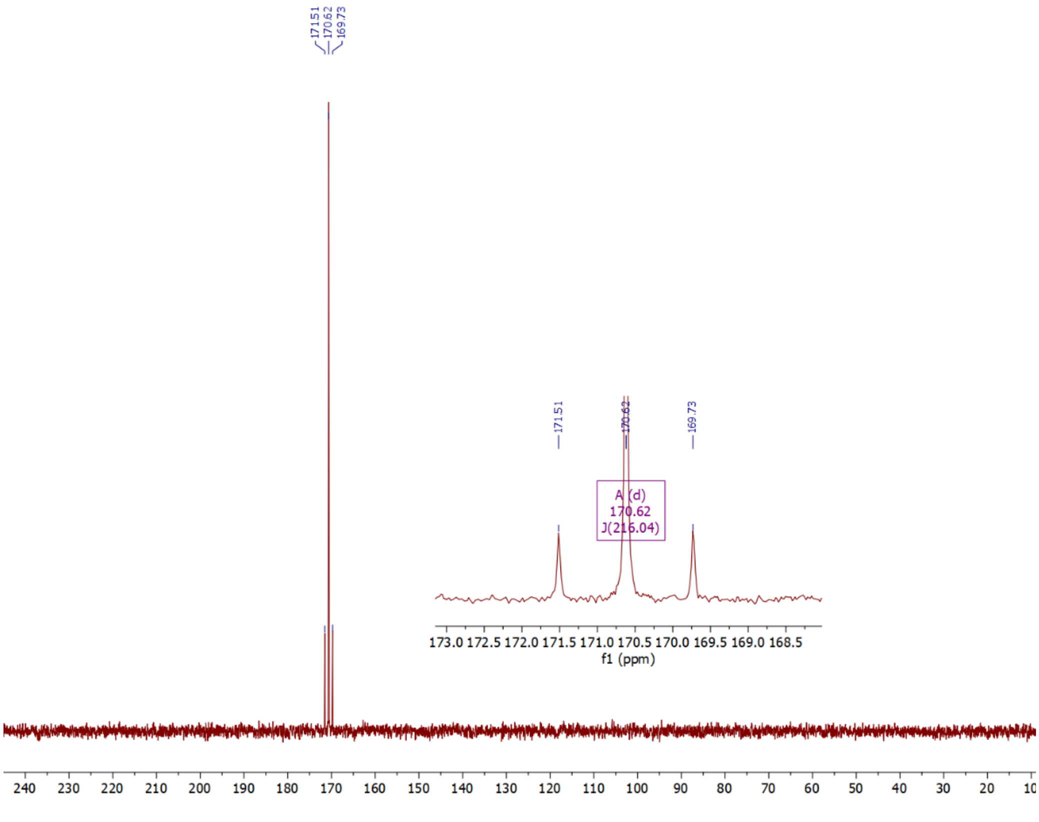

Figure S8. $^{31}P\{^{1}H\}$ NMR spectrum of compound **2** (CD_2Cl_2).

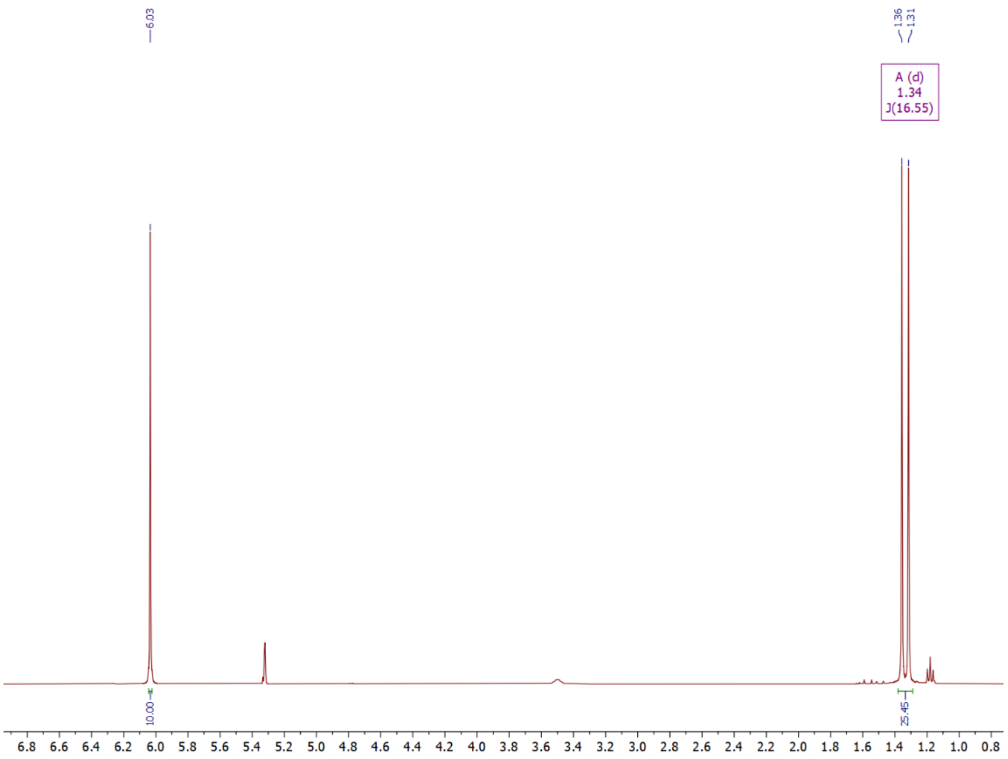

Figure S9. ^{1}H NMR spectrum of compound **2** (CD_2Cl_2).

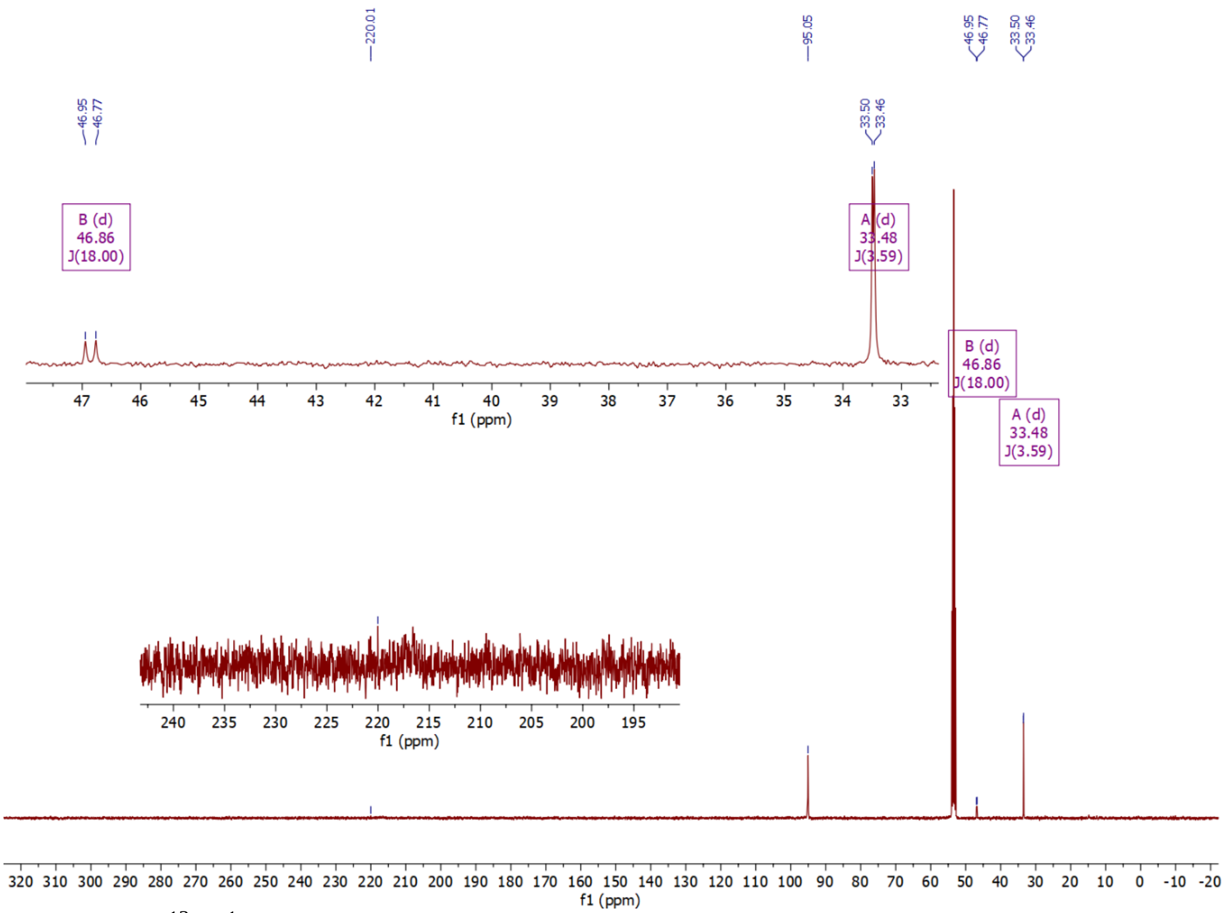

Figure S10. $^{13}C\{^{1}H\}$ NMR spectrum of compound **2** (CD_2Cl_2).

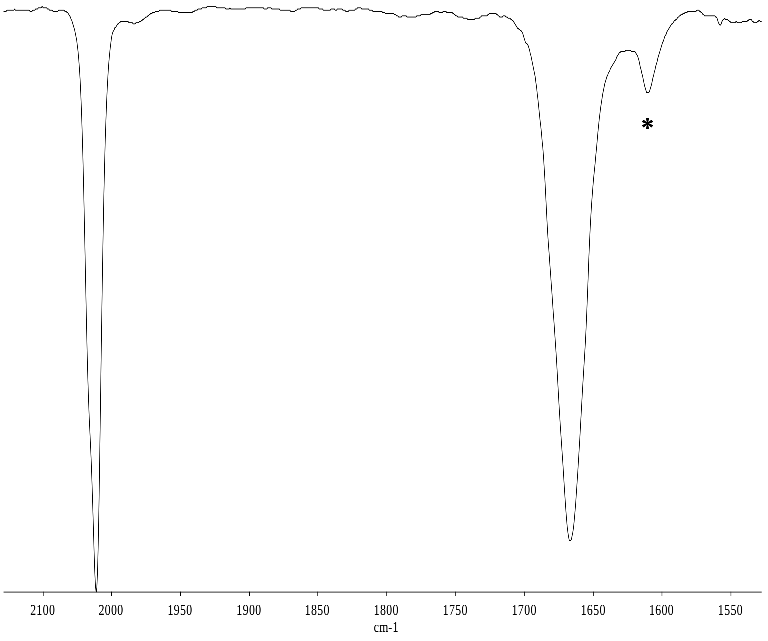

Figure S11. IR spectrum of compound **3** in dichloromethane solution [*= ν_{CC}(Ar)].

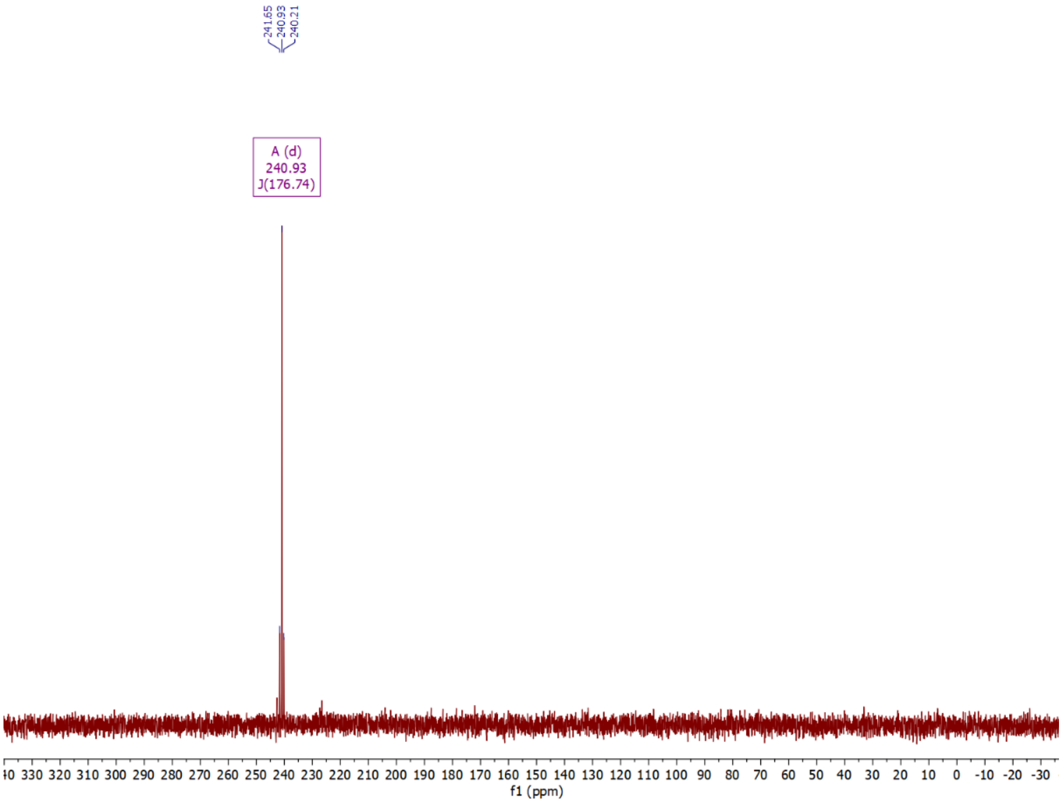

Figure S12. $^{31}P\{^{1}H\}$ NMR spectrum of compound **3** (CD_2Cl_2).

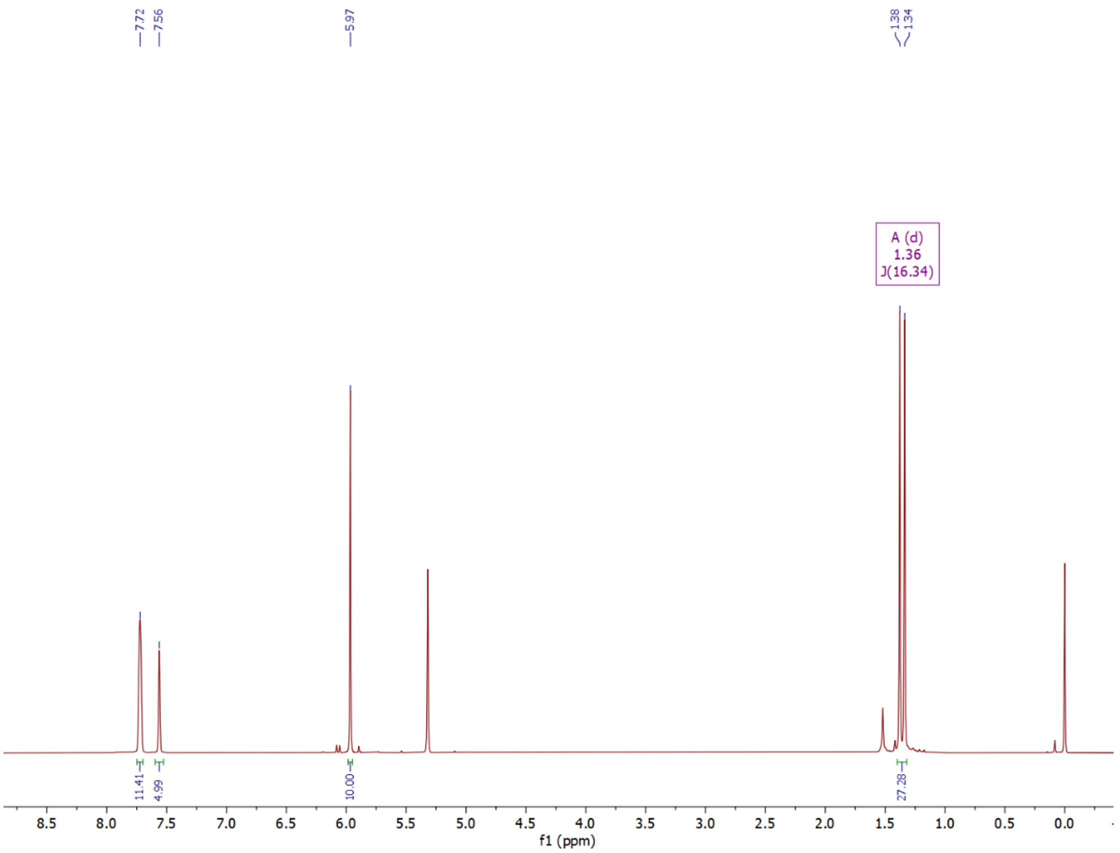

Figure S13. ^{1}H NMR spectrum of compound **3** (CD_2Cl_2).

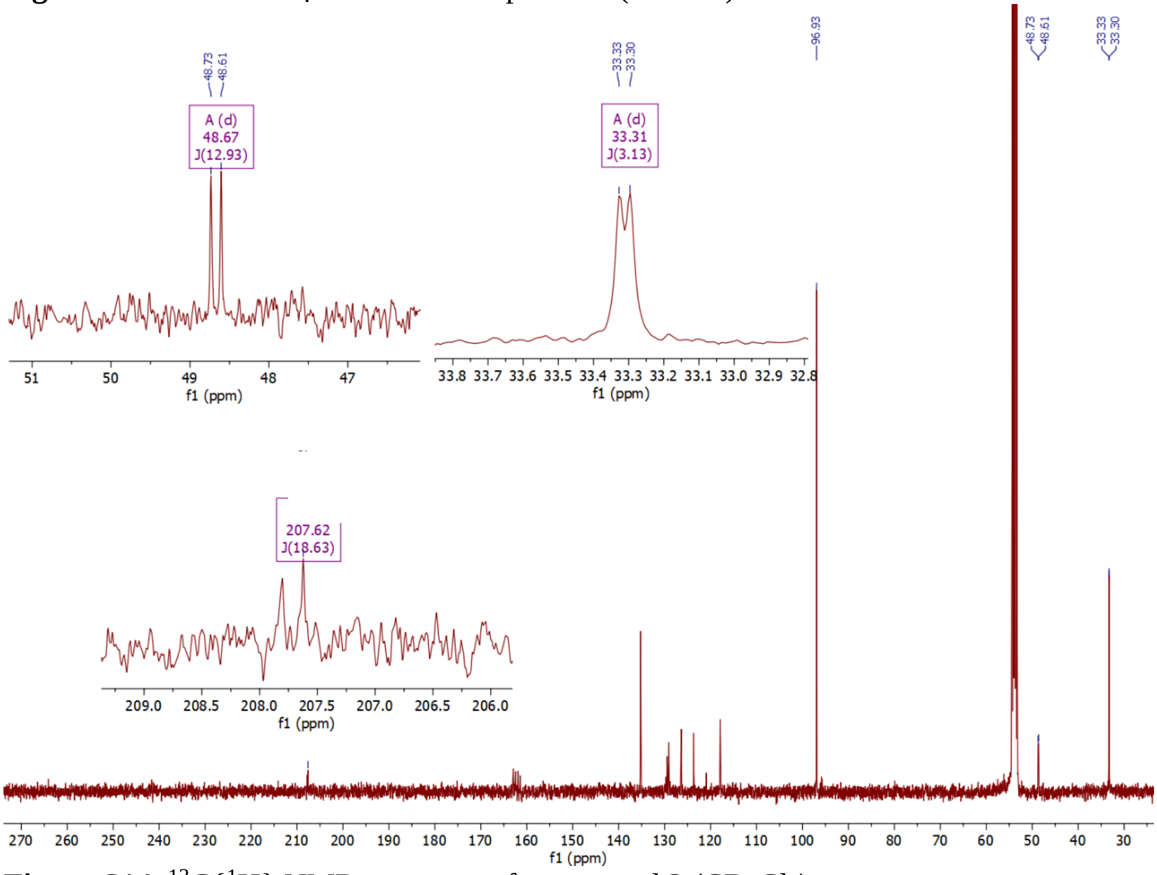

Figure S14. $^{13}C\{^{1}H\}$ NMR spectrum of compound **3** (CD_2Cl_2).

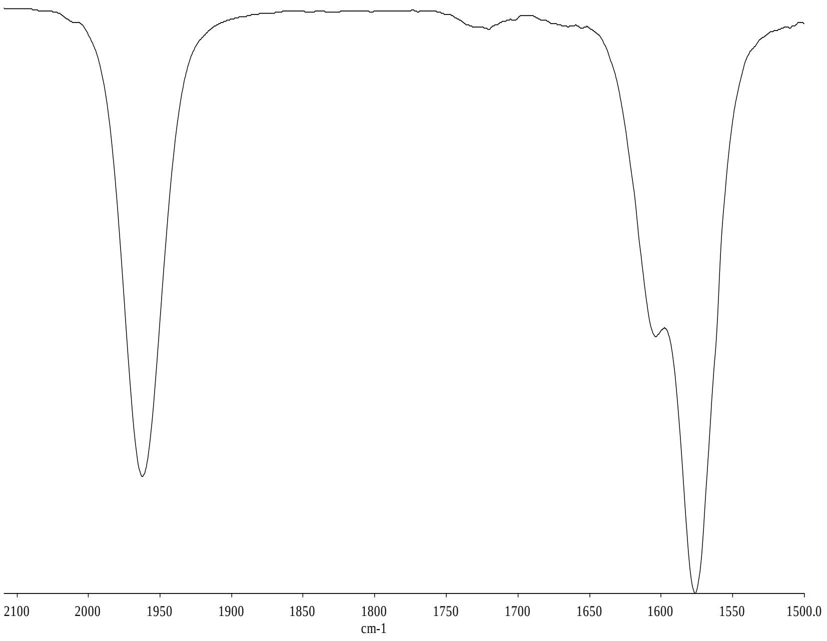

Figure S15. IR spectrum of compound **4** in dichloromethane solution.

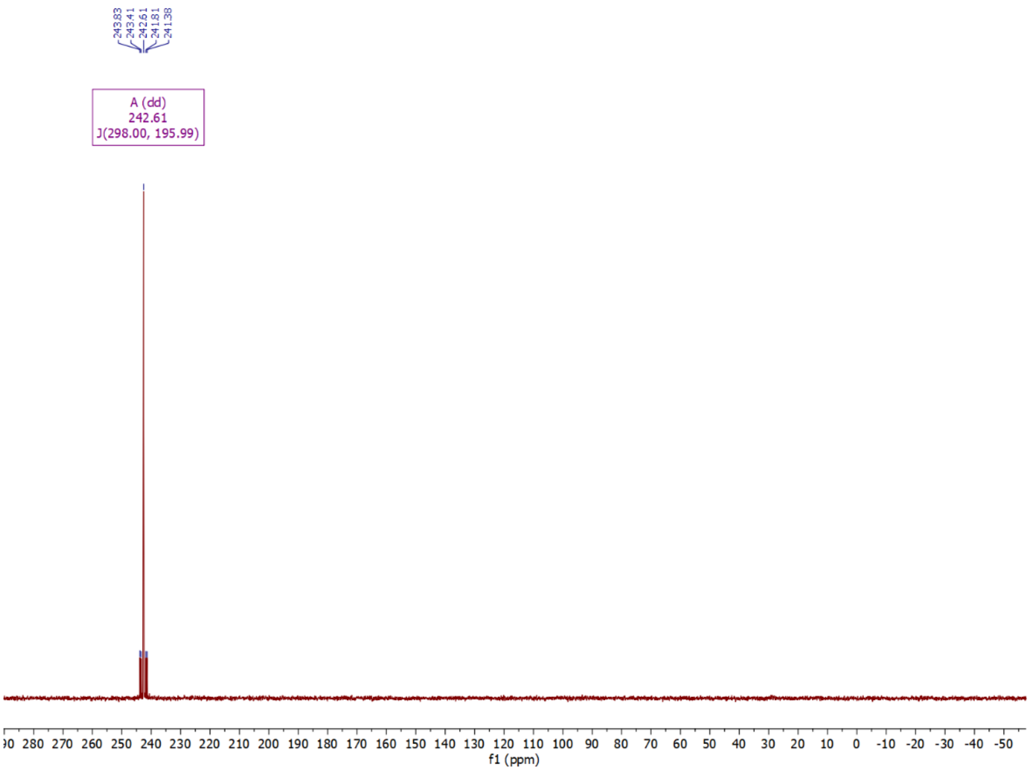

Figure S16. $^{31}P\{^{1}H\}$ NMR spectrum of compound **4** (CD_2Cl_2).

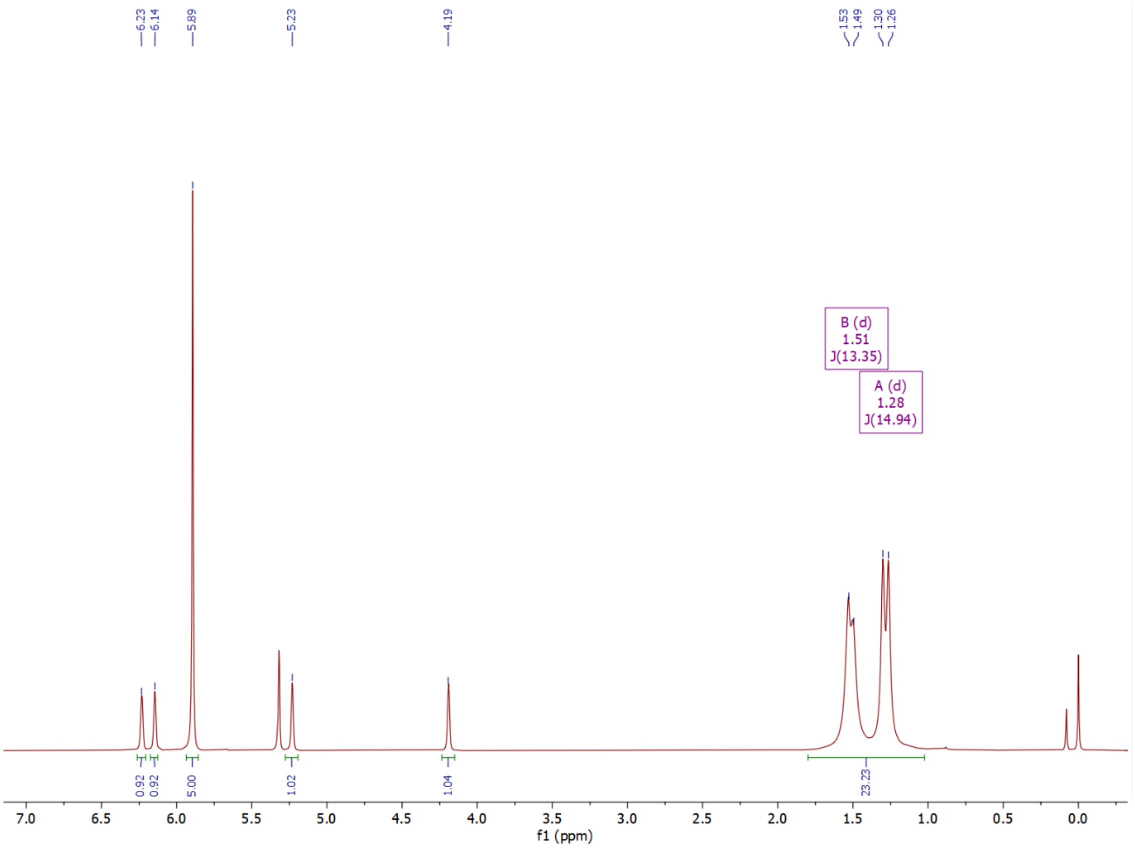

Figure S17. ^{1}H NMR spectrum of compound **4** (CD_2Cl_2).

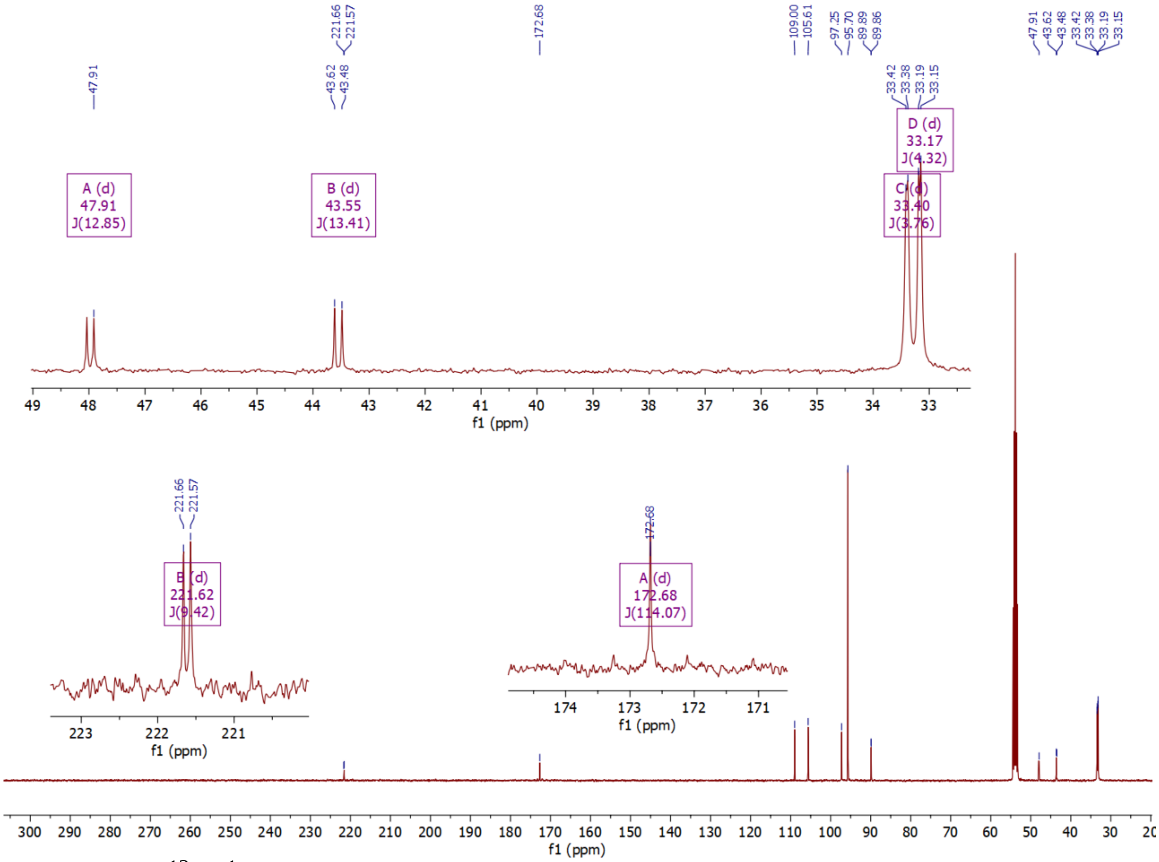

Figure S18. $^{13}C\{^{1}H\}$ NMR spectrum of compound **4** (CD_2Cl_2).

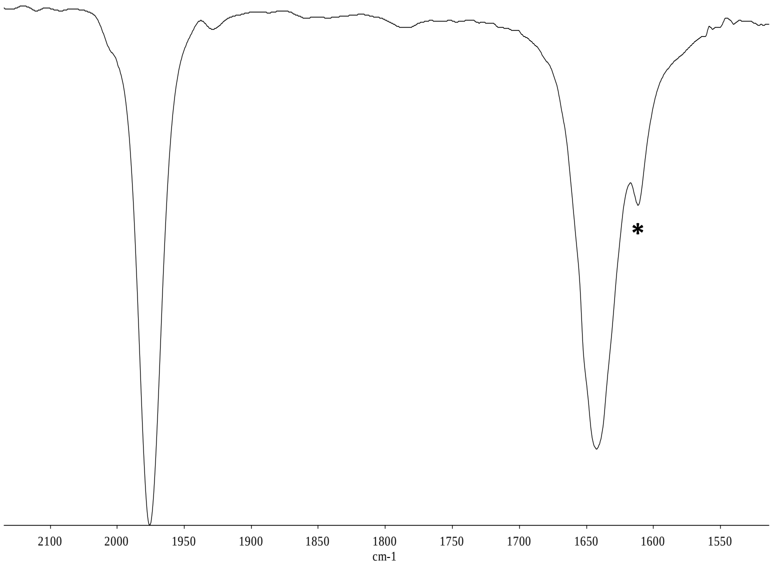

Figure S19. IR spectrum of compound **5** in dichloromethane solution [*= ν_{CC}(Ar)].

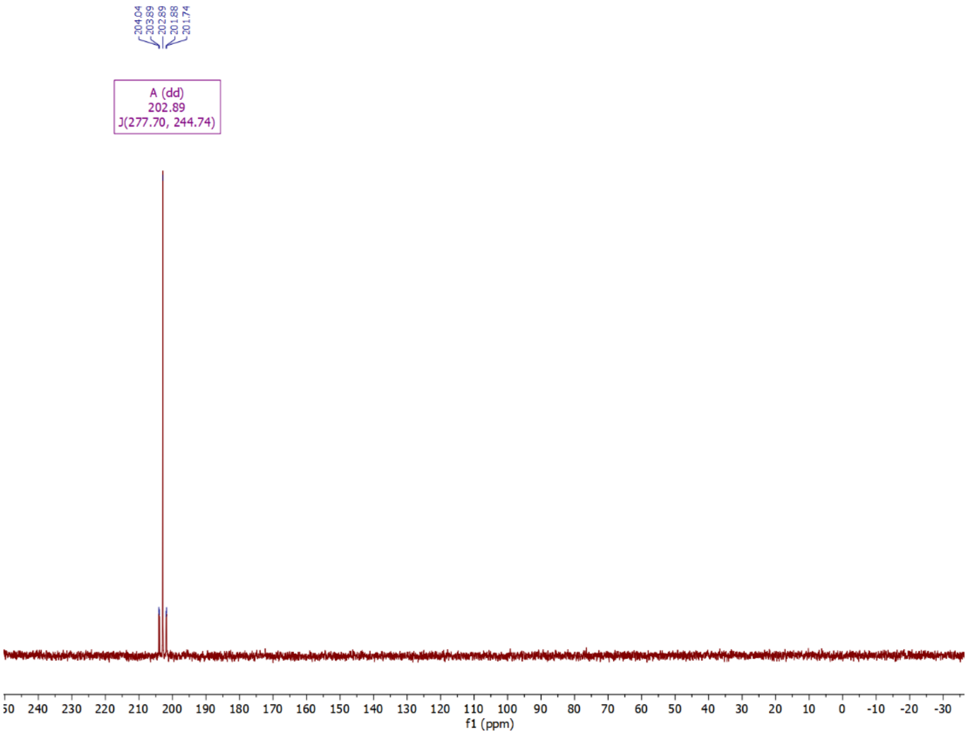

Figure S20. $^{31}P\{^{1}H\}$ NMR spectrum of compound **5** (CD_2Cl_2).

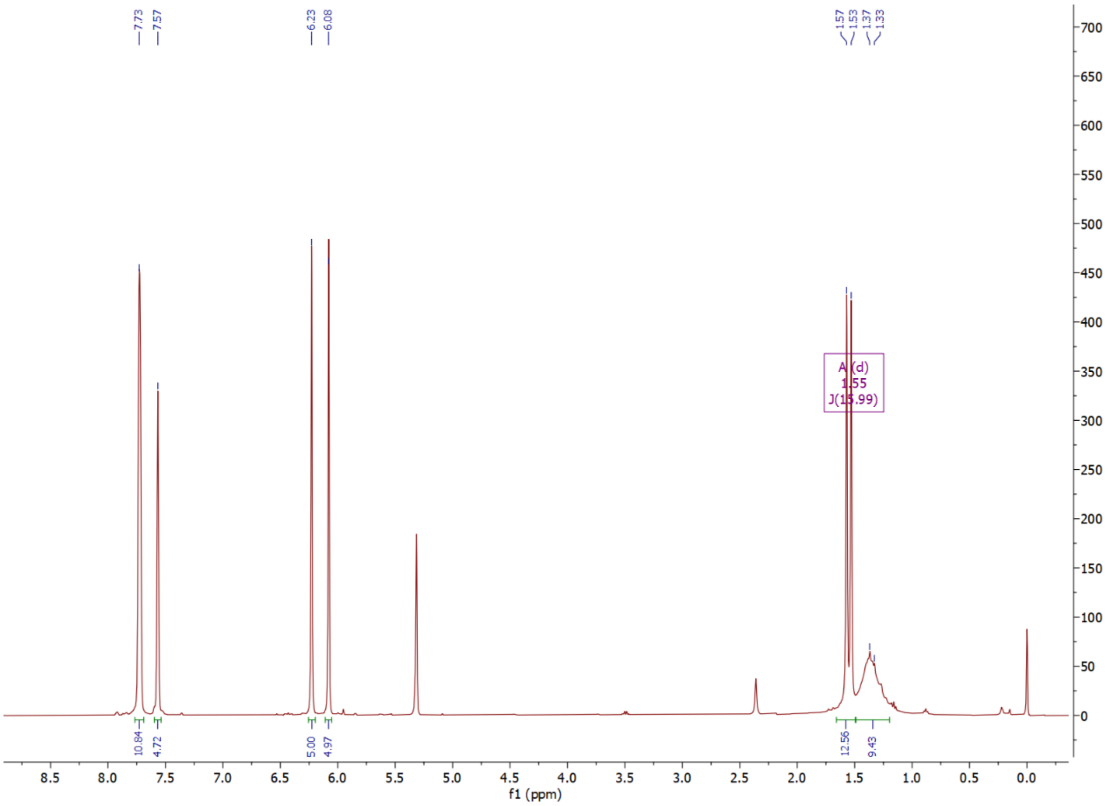

Figure S21. ^{1}H NMR spectrum of compound **5** (CD_2Cl_2).

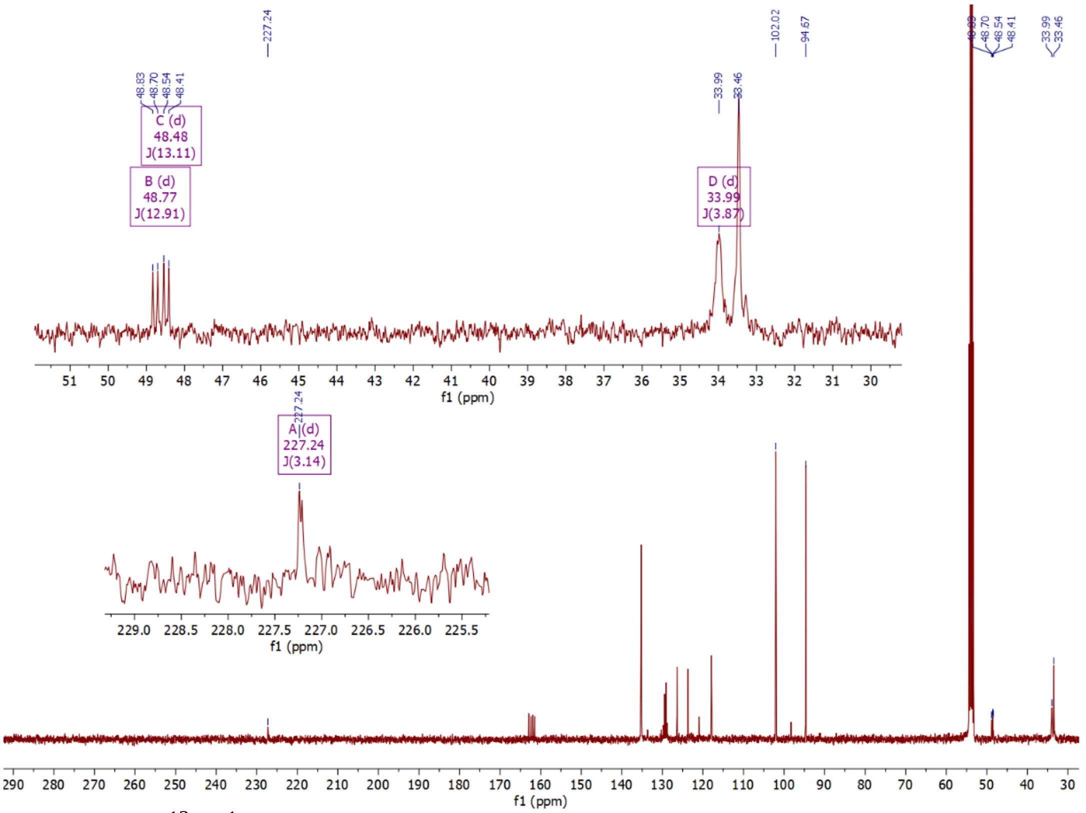

Figure S22. $^{13}C\{^{1}H\}$ NMR spectrum of compound **5** (CD_2Cl_2).

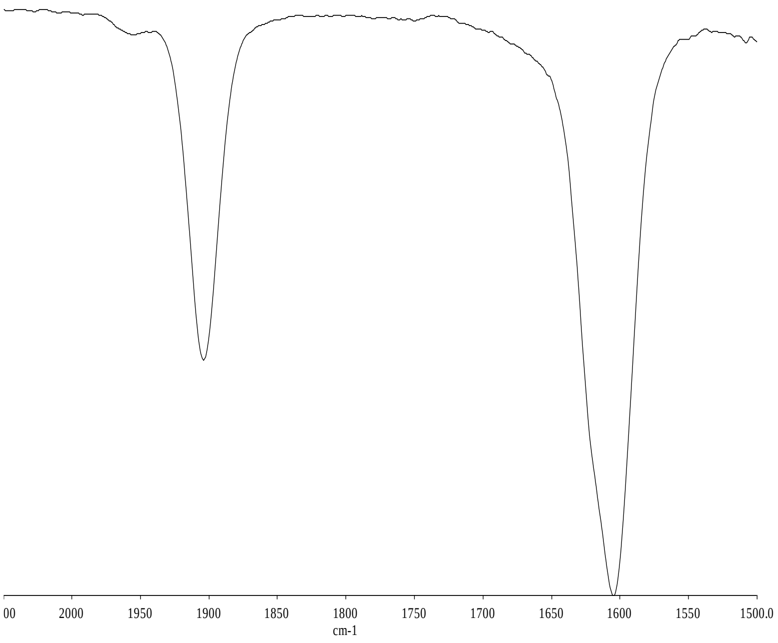

Figure S23. IR spectrum of compound **6** in dichloromethane solution.

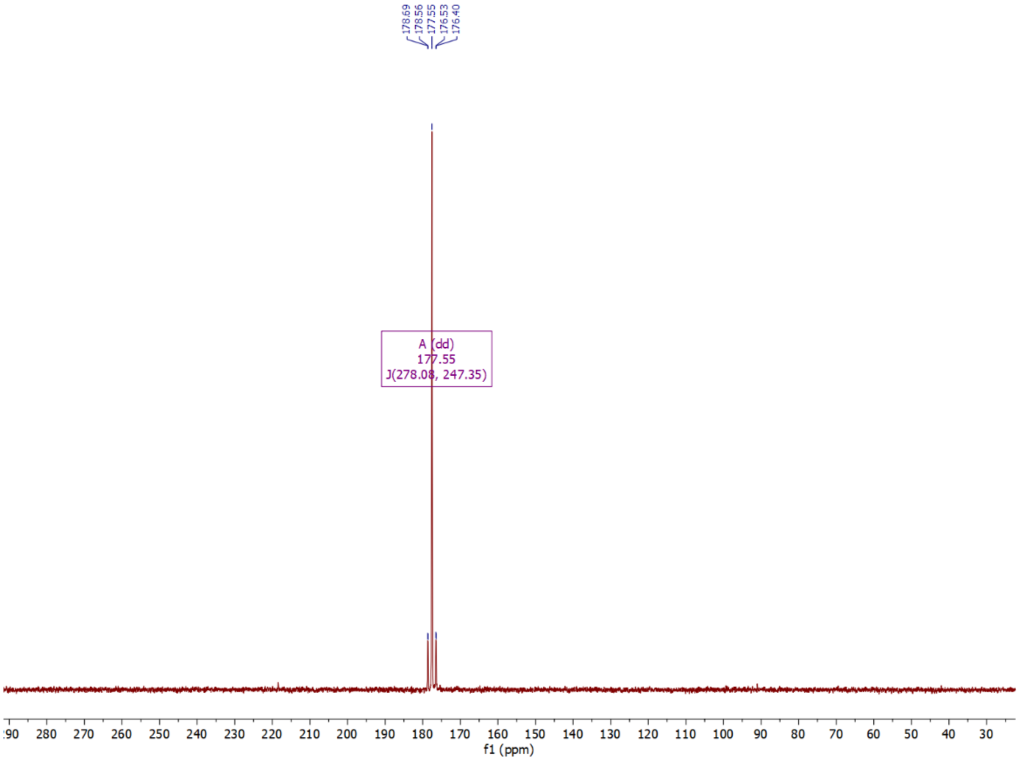

Figure S24. $^{31}P\{^1H\}$ NMR spectrum of compound **6** (CD_2Cl_2).

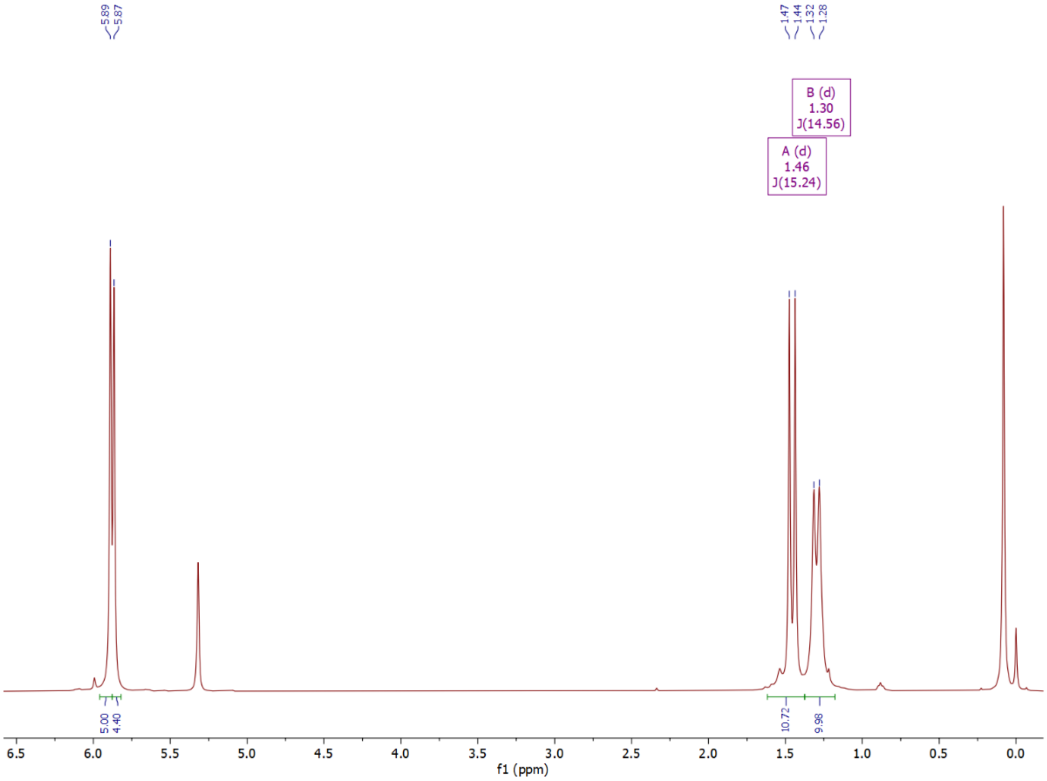

Figure S25. ¹H NMR spectrum of compound **6** (CD_2Cl_2).

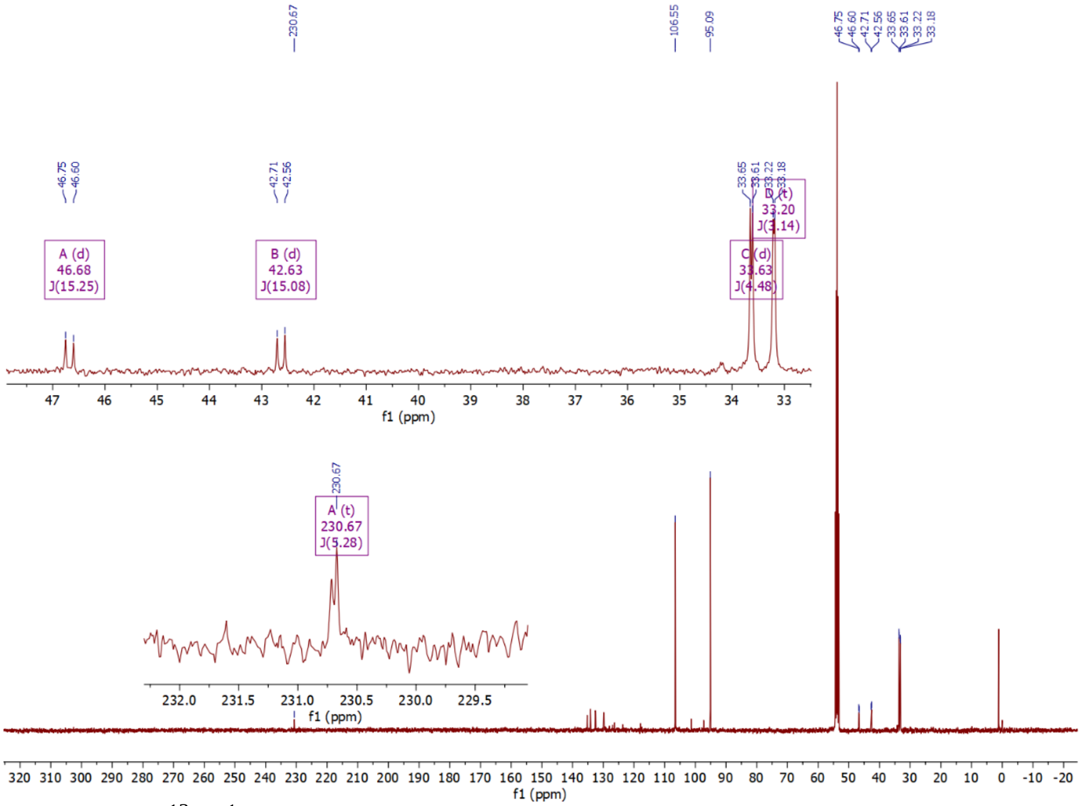

Figure S26. $^{13}C\{^1H\}$ NMR spectrum of compound **6** (CD_2Cl_2).

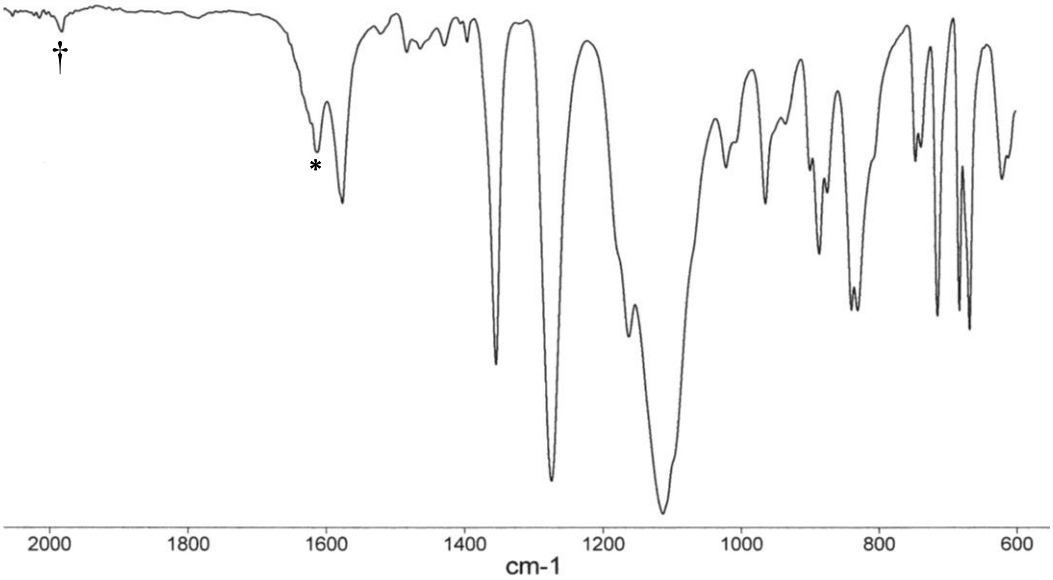

Figure S27. ATR-IR spectrum of a crystalline sample of compound **7** [*= ν_{CC}(Ar)], contaminated with a small amount of the parent compound **5** (†).

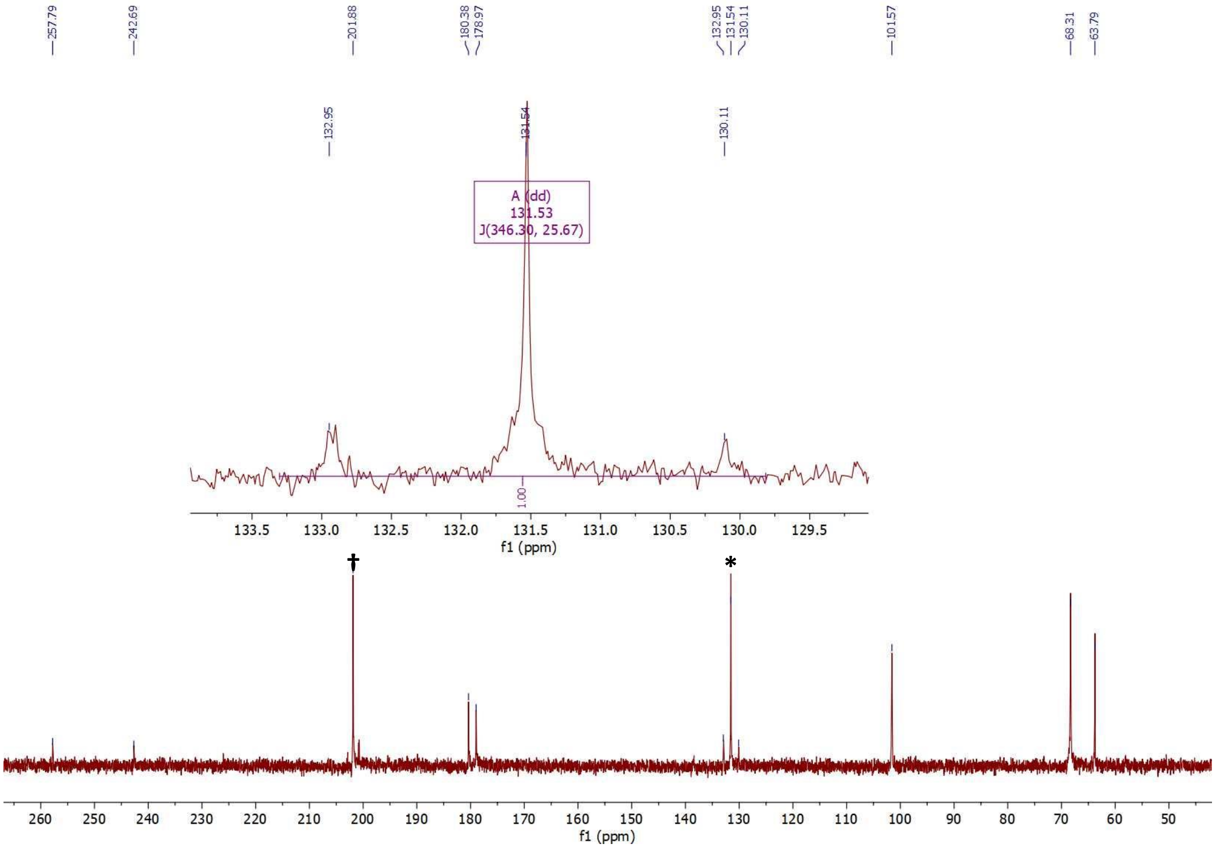

Figure S28. $^{31}P\{^{1}H\}$ NMR spectrum of a crude reaction mixture containing compound **7** (fluorobenzene) [*= compound **7**; †= compound **5**].

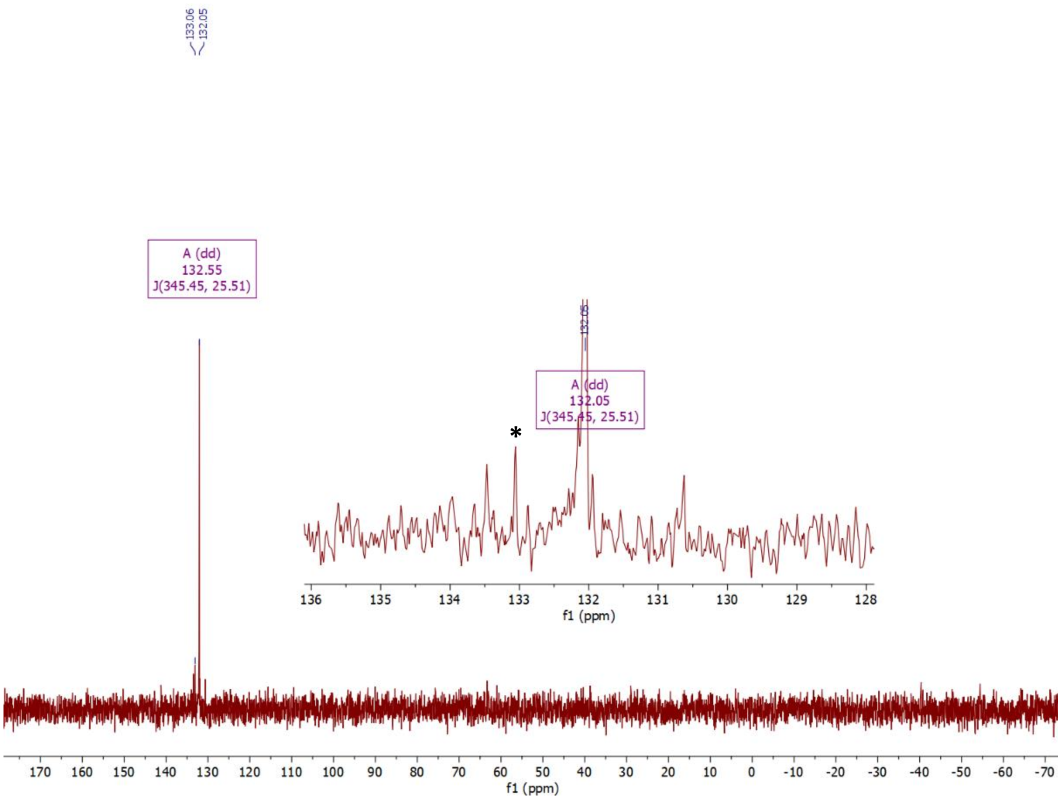

Figure S29. $^{31}P\{^{1}H\}$ NMR spectrum of compound **7** (CD_2Cl_2) [*= unknown impurity].

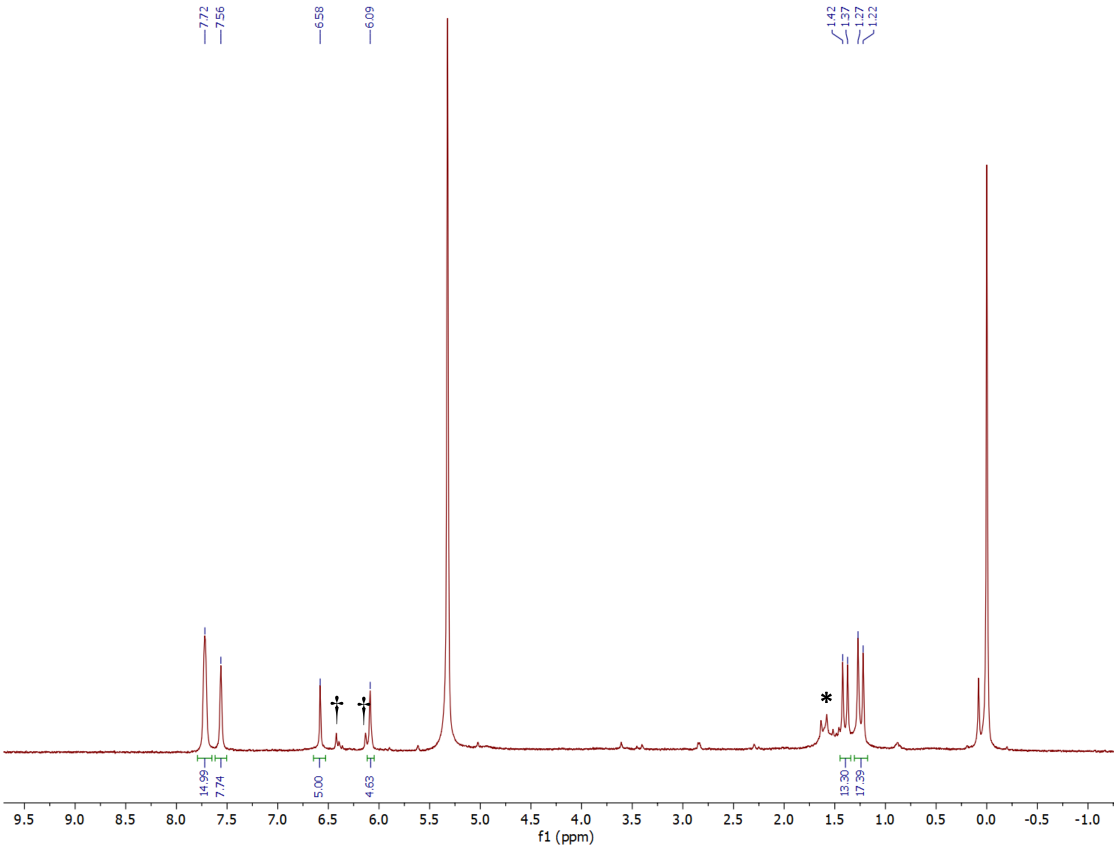

Figure S30. ^{1}H NMR spectrum of compound **7** (CD_2Cl_2), contaminated with a small amount of the parent compound **5** (†). [*= residual water in the solvent].

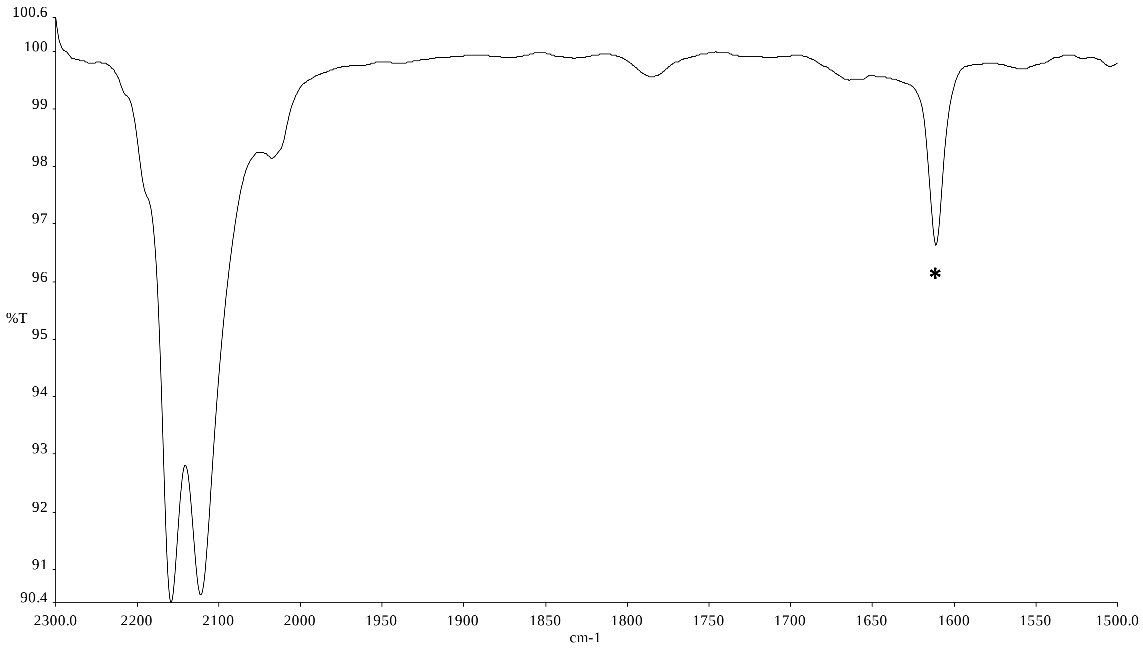

Figure S31. IR spectrum of compound **8** in dichloromethane solution [*= ν_{CC}(Ar)].

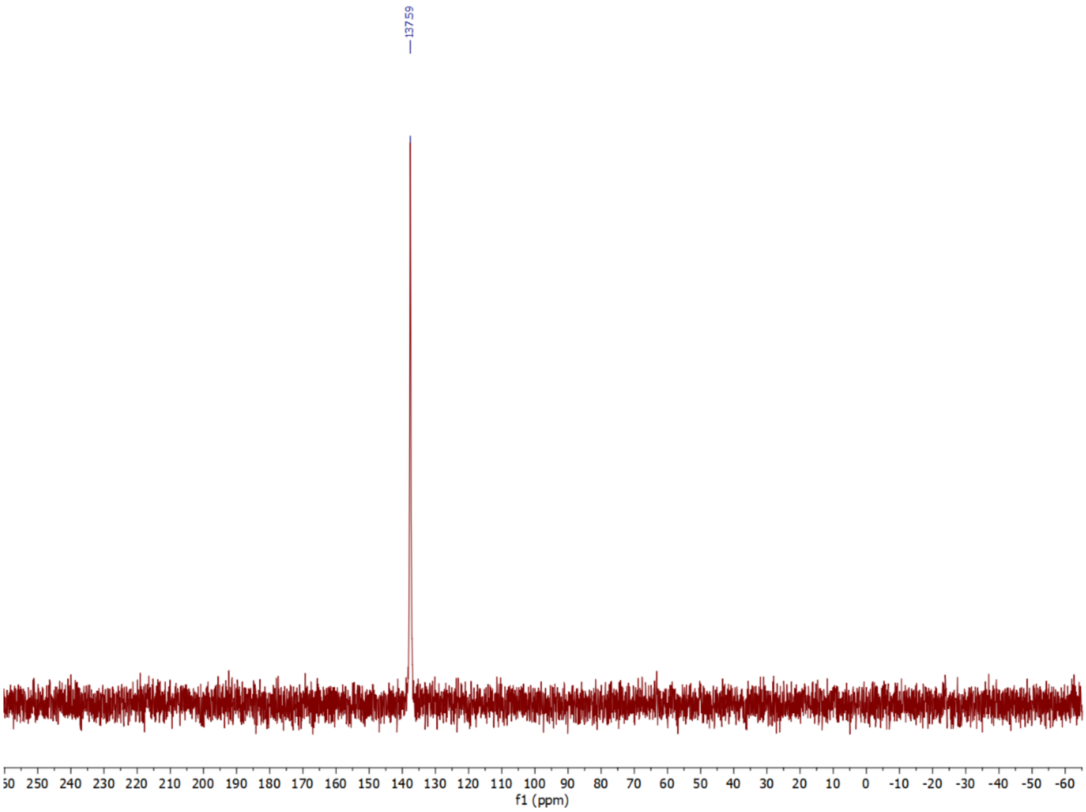

Figure S32. $^{31}P\{^1H\}$ NMR spectrum of compound **8** (CD_2Cl_2).

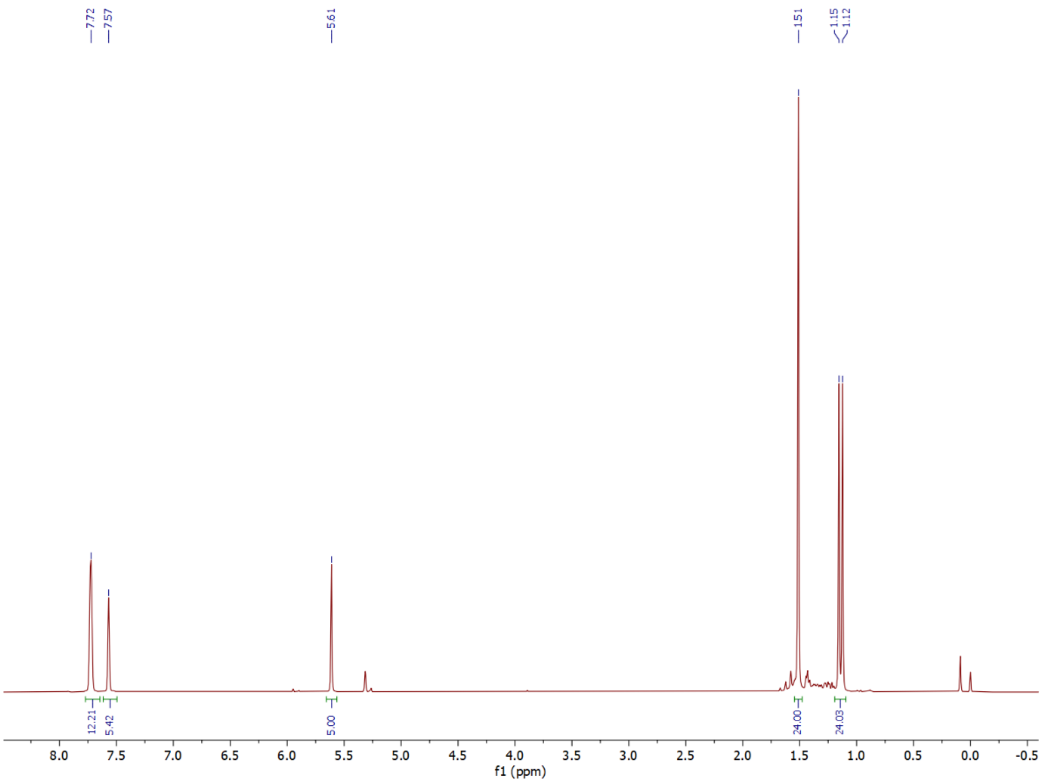

Figure S33. ^{1}H NMR spectrum of compound **8** (CD_2Cl_2).

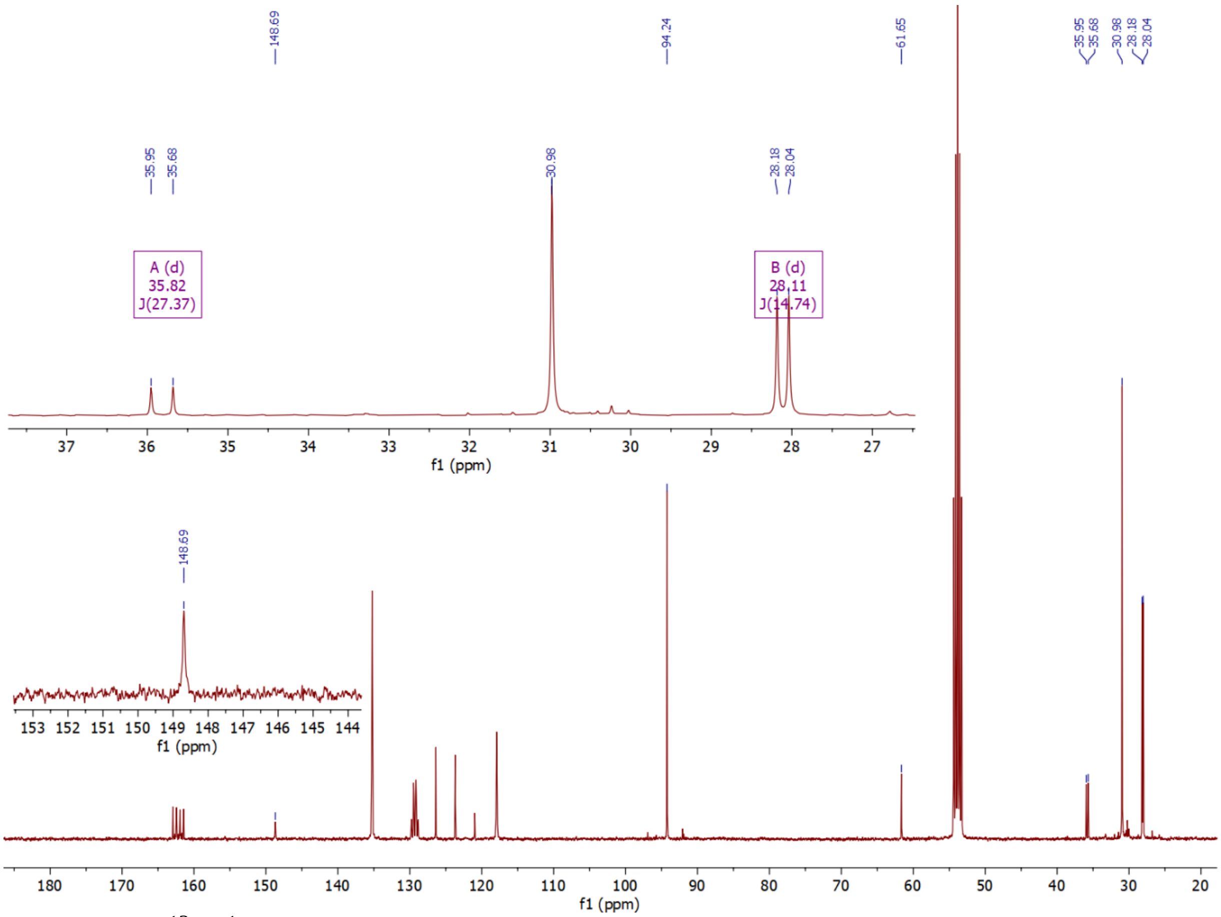

Figure S34. $^{13}C\{^{1}H\}$ NMR spectrum of compound **8** (CD_2Cl_2).

References

1. Armarego, W. L. F.; Chai, C. *Purification of Laboratory Chemicals, 7th ed.*; Butterworth-Heinemann: Oxford, U. K, 2012.
2. Manning, A. R.; Hackett, P.; Birdwhistell, R.; Soye, P. Hexacarbonylbis(η^5-cyclopentadienyl)dichromium, molybdenum, and tungsten, and their analogs, $M_2(\eta^5\text{-}C_5H_4R)_2(CO)_6$ (M = Cr, Mo, W; R = H, Me, or $PhCH_2$). *Inorg. Synth.* **1990**, *28*, 148-150.
3. (*a*) Nishida, H.; Takada, N.; Yoshimura, M.; Sonoda T.; Kobayashi, H. Tetrakis[3,5-bis(trifluoromethyl)phenyl]borate. Highly Lipophilic Stable Anionic Agent for Solvent-extraction of Cations. *Bull. Chem. Soc. Jpn.* **1984**, *57*, 2600-2604. (*b*) Brookhart, M.; Grant B.; Volpe Jr., A. F. $[(3,5\text{-}(CF_3)_2C_6H_3)_4B]^-[H(OEt_2)_2]^+$: a convenient reagent for generation and stabilization of cationic, highly electrophilic organometallic complexes. *Organometallics* **1992**, *11*, 3920-3922.
4. *CrysAlis Pro*; Oxford Diffraction Limited, Ltd.: Oxford, U. K., 2006.
5. Farrugia, L. J. WinGX suite for small-molecule single-crystal crystallography. *J. Appl. Cryst.* **1999**, *32*, 837-838.
6. (a) Sheldrick, G. M. *SHELXL2018*; University of Gottingen, Germany, 2018. (b) Sheldrick, G. M. Crystal structure refinement with SHELXL. *Acta Crystallogr. Sect. C* **2015**, *71*, 3-8. (c) Sheldrick, G. M. A short history of SHELX. *Acta Crystallogr. Sect. A* **2008**, *64*, 112-122.
7. *APEX4 v2021.10-0*; Bruker AXS Inc.: Madison (WI), USA, 2021.
8. *SAINT v8.40B*; Bruker AXS Inc.: Madison (WI), USA, 2018.
9. Krause, L.; Herbst-Irmer, R.; Sheldrick G.M.; Stalke D. Comparison of silver and molybdenum microfocus X-ray sources for single-crystal structure determination *J. Appl. Cryst.* **2015**, *48*, 3-10.
10. *CrysAlisPro v1.171.39.46*; Rigaku OD: Austin (TX), USA, 2018.
11. Sheldrick, G. M. SHELXT – Integrated space-group and crystal-structure determination. *Acta Crystallogr. Sect. A*, **2015**, 71, 3-8.
12. Spek, A. L. PLATON SQUEEZE: a tool for the calculation of the disordered solvent contribution to the calculated structure factors. *Acta Crystallogr. Sect. C* **2015,** *71*, 9-18.
13. Boyle, P. D. COSET: a program for deriving and testing merohedral and pseudo-merohedral twin laws. *J. Appl. Cryst.* **2014**, *47*, 467-470.
14. Flack, H. D. The derivation of twin laws for (pseudo-)merohedry by coset decomposition *Acta Crystallogr. Sect. A*, **1987**, *43*, 564-568.
15. Dolomanov, O. V.; Bourhis, L. J.; Gildea, R. J.; Howard, J. A. K.; Puschmann, H. OLEX2: a complete structure solution, refinement and analysis program *J. Appl. Cryst.* **2009**, *42*, 339-341.
16. *Olex2 v1.5* © 2004 – 2022; OlexSys Ltd.: Durham, UK, 2022.
17. Frisch, M. J.; Trucks, G. W.; Schlegel, H. B.; Scuseria, G. E.; Robb, M. A.; Cheeseman, J. R.; Scalmani, G.; Barone, V.; Petersson, G. A.; Nakatsuji, H.; Li, X.; Caricato, M.; Marenich, A. V.; Bloino, J.; Janesko, B. G.; Gomperts, R.; Mennucci, B.; Hratchian, H. P.; Ortiz, J. V.; Izmaylov, A. F.; Sonnenberg, J. L.; Williams-Young, D.; Ding, F.; Lipparini, F.; Egidi, F.; Goings, J.; Peng,

B.; Petrone, A.; Henderson, T.; Ranasinghe, D.; Zakrzewski, V. G.; Gao, J.; Rega, N.; Zheng, G.; Liang, W.; Hada, M.; Ehara, M.; Toyota, K.; Fukuda, R.; Hasegawa, J.; Ishida, M.; Nakajima, T.; Honda, Y.; Kitao, O.; Nakai, H.; Vreven, T.; Throssell, K.; Montgomery, J. A., Jr.; Peralta, J. E.; Ogliaro, F.; Bearpark, M. J.; Heyd, J. J.; Brothers, E. N.; Kudin, K. N.; Staroverov, V. N.; Keith, T. A.; Kobayashi, R.; Normand, J.; Raghavachari, K.; Rendell, A. P.; Burant, J. C.; Iyengar, S. S.; Tomasi, J.; Cossi, M.; Millam, J. M.; Klene, M.; Adamo, C.; Cammi, R.; Ochterski, J. W.; Martin, R. L.; Morokuma, K.; Farkas, O.; Foresman, J. B.; Fox, D. J. *Gaussian 16, Revision A.03*; Gaussian, Inc.: Wallingford, CT, USA, 2016.

18. Zhao Y.; Truhlar, D. G. A new local density functional for main-group thermochemistry, transition metal bonding, thermochemical kinetics, and noncovalent interactions. *J. Chem. Phys.* **2006**, *125,* 194101: 1-18.
19. Grimme, S.; Antony, J.; Ehrlich, S.; Krieg, H. A consistent and accurate ab initio parameterization of density functional dispersion correction (DFT-D) for the 94 elements H-Pu. *J. Chem. Phys.* **2010**, *132*, 154104.
20. Hay, P. J.; Wadt, W. R. Ab initio effective core potentials for molecular calculations. Potentials for potassium to gold including the outermost core orbitals. *J. Chem. Phys.* **1985**, *82*, 299-310.
21. (a) Hariharan, P. C.; Pople, J. A. Influence of polarization functions on MO hydrogenation energies. *Theor. Chim. Acta* **1973**, *28*, 213-222. (b) Petersson, G. A.; Al-Laham, M. A. A complete basis set model chemistry. II. Open-shell systems and the total energies of the first-row atoms. *J. Chem. Phys.* **1991**, *94*, 6081-6090. (c) Petersson, G. A.; Bennett, A.; Tensfeldt, T. G.; Al-Laham, M. A.; Shirley, W. A.; Mantzaris, J. A complete basis set model chemistry. I. The total energies of closed-shell atoms and hydrides of the first-row elements. *J. Chem. Phys.* **1988**, *89*, 2193-2218.
